# Kir2.1 dysfunction at the sarcolemma and the sarcoplasmic reticulum causes arrhythmias in a mouse model of Andersen–Tawil syndrome type 1

**DOI:** 10.1038/s44161-022-00145-2

**Published:** 2022-10-17

**Authors:** Álvaro Macías, Andrés González-Guerra, Ana I. Moreno-Manuel, Francisco M. Cruz, Lilian K. Gutiérrez, Nieves García-Quintáns, Marta Roche-Molina, Francisco Bermúdez-Jiménez, Vicente Andrés, María Linarejos Vera-Pedrosa, Isabel Martínez-Carrascoso, Juan A. Bernal, José Jalife

**Affiliations:** 1 Centro Nacional de Investigaciones Cardiovasculares (CNIC), Madrid, Spain; 2 CIBER de Enfermedades Cardiovasculares (CIBERCV), Madrid, Spain; 3 Departments of Medicine and Molecular and Integrative Physiology, University of Michigan, Ann Arbor, MI USA

**Keywords:** Cell biology, Arrhythmias

## Abstract

Andersen–Tawil syndrome type 1 (ATS1) is associated with life-threatening arrhythmias of unknown mechanism. In this study, we generated and characterized a mouse model of ATS1 carrying the trafficking-deficient mutant Kir2.1^Δ314-315^ channel. The mutant mouse recapitulates the electrophysiological phenotype of ATS1, with QT prolongation exacerbated by flecainide or isoproterenol, drug-induced QRS prolongation, increased vulnerability to reentrant arrhythmias and multifocal discharges resembling catecholaminergic polymorphic ventricular tachycardia (CPVT). Kir2.1^Δ314-315^ cardiomyocytes display significantly reduced inward rectifier K^+^ and Na^+^ currents, depolarized resting membrane potential and prolonged action potentials. We show that, in wild-type mouse cardiomyocytes and skeletal muscle cells, Kir2.1 channels localize to sarcoplasmic reticulum (SR) microdomains, contributing to intracellular Ca^2+^ homeostasis. Kir2.1^Δ314-315^ cardiomyocytes exhibit defective SR Kir2.1 localization and function, as intact and permeabilized Kir2.1^Δ314-315^ cardiomyocytes display abnormal spontaneous Ca^2+^ release events. Overall, defective Kir2.1 channel function at the sarcolemma and the SR explain the life-threatening arrhythmias in ATS1 and its overlap with CPVT.

## Abstract (web summary)

Macias et al. generated a mouse model recapitulating the electrophysiological features of Andersen–Tawil syndrome type 1 (ATS1), a disease associated with life-threatening arrhythmias, via AAV-mediated in vivo expression of trafficking-deficient mutant Kir2.1^Δ314-315^ channel. The authors identify functional Kir2.1 receptors in sarcoplasmic reticulum (SR) microdomains and report that the ATS1 arrhythmogenic phenotype is due to the dysfunction of Kir2.1 receptors both in the sarcolemma and in the SR domains.

## Main

Trafficking-deficient mutations in the gene coding the strong inward rectifier K^+^ channel Kir2.1 (*KCNJ2*) result in autosomal-dominant Andersen–Tawil syndrome type 1 (ATS1) (refs. ^1,2^). ATS1 is manifested as a triad of ventricular arrhythmias, periodic paralysis and dysmorphic features^3,4^. Its electrocardiographic manifestations include QT prolongation, ventricular ectopy, bigeminy, polymorphic or bidirectional ventricular tachycardia and sudden cardiac death (SCD)^5^. In some ‘atypical cases’, ATS1 overlaps phenotypically with catecholaminergic polymorphic ventricular tachycardia (CPVT)^5,6^. Kir2.1 is the main channel controlling both the resting membrane potential and the final phase of ventricular repolarization^7^. Kir2.1 forms channelosomes with the main cardiac voltage-gated Na^+^ channel (Na_V_1.5) that help to control ventricular excitability and propagation velocity^7,8^. Emerging evidence indicates that these two widely different channels physically interact from early stages of protein assembly and trafficking and share common partners that may include, but are not limited to, anchoring/adapter proteins, enzymes and scaffolding and regulatory proteins^8–12^, highlighting the relevance of macromolecular ion channel interplay in cardiac diseases^13^.

In vitro approaches have demonstrated that Na_V_1.5 and Kir2.1 proteins co-localize at the sarcolemma and regulate each other’s levels via PDZ-mediated binding to either the MAGUK protein SAP97 or α1-syntrophin, both of which help to stabilize both channels^8,9^. Moreover, co-expression of trafficking-deficient mutant Kir2.1^Δ314-315^ channels with wild-type (WT) Na_V_1.5 reduces both *I*_*K1*_ and *I*_*Na*_, suggesting that the Na_V_1.5–Kir2.1 complex pre-assembles early in the forward trafficking pathway and that both channels traffic more efficiently as the Na_V_1.5–Kir2.1 complex than alone^9,10,14^. However, it is unknown whether trafficking-deficient mutations in vivo affect Na_V_1.5–Kir2.1 interactions, leading to reduced excitability and ventricular arrhythmias severe enough to result in SCD. Also unknown is the mechanism by which some *KCNJ2* mutation carriers present arrhythmias resembling CPVT.

Two different pathways^10,15^ have been described through which Kir2.1 and Na_V_1.5 can traffic together or individually, and the possibility exists that either Kir2.1 or Na_V_1.5, or both, remain trapped at the Golgi or the sarcoplasmic reticulum (SR) where they might be functional. Here we tested the hypothesis that, in addition to reduced *I*_*K1*_, a proportion of patients with ATS1 have reduced *I*_*Na*_ in the atria and ventricles, because of trafficking disruption of the macromolecular complex that includes Kir2.1 and Na_V_1.5, leading to conduction defects and arrhythmias. Moreover, we present evidence indicating that, in addition to Kir2.1 channels that co-localize with Na_V_1.5 at the sarcolemma, a unique population of Kir2.1 channels retained at the SR may help to maintain intracellular Ca^2+^ homeostasis and excitation–contraction (e–c) coupling. If oriented as we surmise, SR Kir2.1 channels should underlie a fundamental countercurrent for SR Ca^2+^-ATPase (SERCA)-mediated Ca^2+^ re-uptake to the ryanodine receptor (RyR)-mediated Ca^2+^ release. In addition, we demonstrate herein that defects in SR Kir2.1 localization and function contribute to abnormal spontaneous Ca^2+^ release in ATS1 in both intact and permeabilized cardiomyocytes. Altogether, our analysis reveals a dual molecular mechanism at the sarcolemma and the SR membrane for the characteristic life-threatening arrhythmias in patients with ATS1 and how SR Kir2.1 channel dysfunction contributes to the phenotypic overlap between ATS1 and CPVT.

## Results

### Kir2.1^Δ314-315^ is a trafficking-deficient protein in vivo

**Fig. 1:**
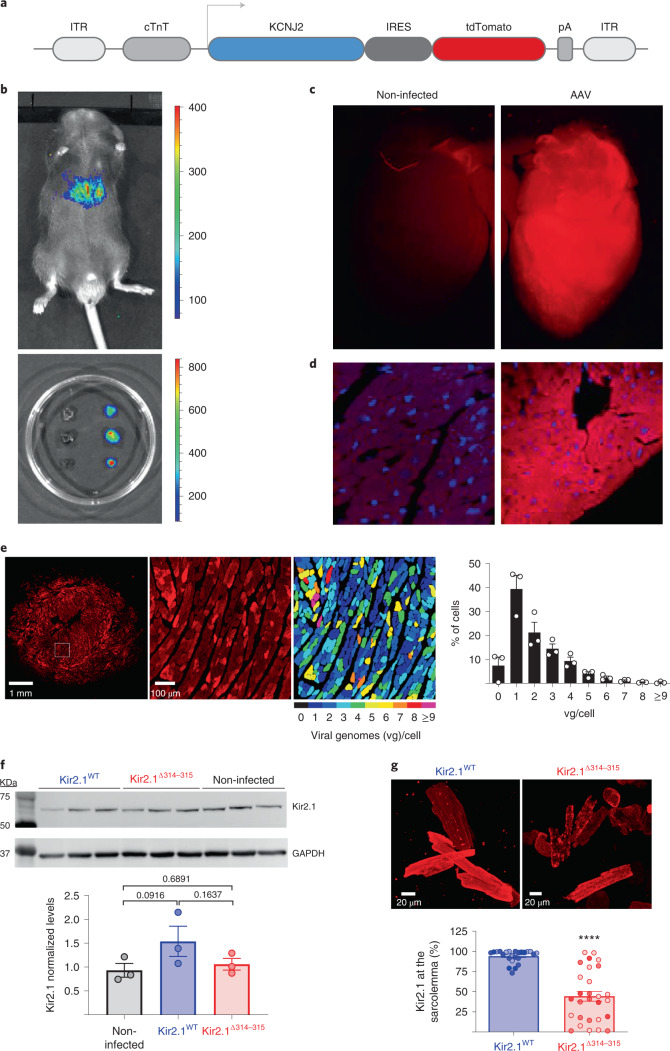
AAV-mediated in vivo expression of Kir2.1^Δ314-315^. **a**, AAV-based vector encoding human *KCNJ2* (Kir2.1 channel), WT or Δ314-315 mutant, driven by the *cTnT* proximal promoter, followed by enhanced *tdTomato* after an IRES. **b**, Imaging of luciferase transgene expression under *cTnT* cardiospecific control comparing AAV-infected and non-infected mice. Upper, live C57BL/6J mouse injected (femoral vein) with AAV–*Luciferase* control vector in packaging serotype 9 (dose 3.5 × 10^10^ vg in 50 µl of saline solution). Lower, three isolated hearts from *Luciferase-*injected animals are shown next to three hearts from non-infected mice (saline solution). Images taken 4 weeks after inoculation. **c**,**d**, Representative fluorescence images of two non-injected (left) and AAV-Kir2.1^∆314-315^ transduced (right) mice, the latter showing expression of tdTomato throughout the heart (*n* = 5 per group). **e**, Left, representative fluorescence microscopy images of short-axis cross-sections of AAV-transduced hearts illustrate expression of tdTomato throughout the heart. Middle, a cropped image used for quantification. Scale bar, 100 μm. Right, fluorescence intensity staining and quantification (*n* = 3 mice, *n* = 1,465 cells) of transduced protein expression, used to assign the number of integrated viral genomes per cardiomyocyte. **f**, Representative western blots and quantification of total and membrane protein extracts from control and AAV-transduced isolated cardiomyocytes. Levels of expression were corrected by GAPDH expression and normalized by non-injected levels. Statistical analyses were conducted using two-tailed Mann–Whitney *t*-test. **g**, Representative immunostaining pattern of Kir2.1 at the plasma membrane in isolated cardiomyocytes from Kir2.1^WT^-expressing and Kir2.1^∆314-315^-expressing mice. Graph shows the percentage of cell surface with positive staining. Different colors in the same group identify cells coming from one animal. Statistical analyses were conducted using two-level hierarchical *t*-test analysis followed by Bonferroni’s post test. Each value is represented as mean ± s.e.m. ****P < 0.0001. Source data

**Extended Data Fig. 1:**
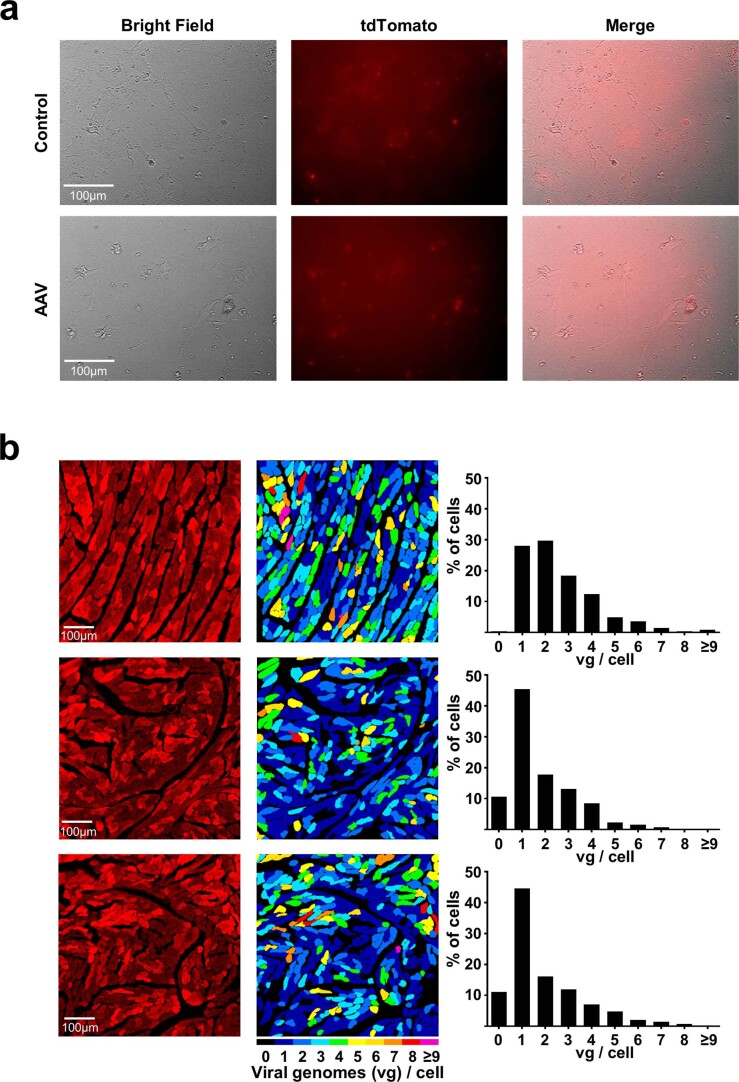
AAV-mediated KCNJ2 expression is specific and homogenous. AAV-*KCNJ2*^*Δ314-315*^ associated with the *cTn*T promoter is not expressed in cardiac fibroblasts. Representative fluorescence images of cardio-fibroblasts isolated from control and AAV-transduced mice to compare expression levels of tdTomato in non-cardiomyocyte cells. **b**, Quantitative analysis of viral genomes present in cardiomyocytes of infected mouse hearts. *Left*, representative fluorescence microscopy images of short-axis cross sections of 3 different AAV-transduced hearts illustrate expression of tdTomato throughout the heart. *Middle*, images used for quantification. *Right*, quantification of transduced protein expression, used to assign the number of integrated viral genomes (vg) per cardiomyocyte.

Using intravenous (i.v.) adeno-associated virus (AAV)-mediated gene transfer, we generated homogeneous cohorts of mice expressing the WT *KCNJ2* gene coding the inward rectifier K^+^ channel Kir2.1, Kir2.1^WT^ and a *KCNJ2* gene variant coding a trafficking-deficient protein with an internal deletion (Δ314-315)^10,16^ called Kir2.1^Δ314-315^ (Fig. 1a). We achieved cardiomyocyte-specific gene expression in live mice using AAV serotype 9 gene delivery in combination with shuttle constructs expressing target genes under the transcriptional control of the troponin-T proximal promoter (*cTnT)* as described^17^. Test mice transduced with a reporter construct encoding Luciferase were used to demonstrate specific and homogeneous distribution throughout the ventricles (Fig. 1b). Using the same cardio-specific shuttle AAV vector, we expressed in vivo WT Kir2.1 (*Kir2.1*^*WT*^) or *Kir2.1*^*Δ314-315*^ mutant followed by enhanced tandem dimer tomato (tdTomato) fluorescence protein after an internal ribosome entry site (IRES). A single i.v. injection of AAV resulted in long-lasting and homogeneous gene transexpression in cardiomyocytes (Fig. 1c,d) without detectable expression in other cellular heart components, such as fibroblasts (Extended Data Fig. 1a). Efficiency of cardiomyocyte transduction after viral infection was measured by tdTomato immunofluorescence. Quantitative analysis revealed that more than 90% of cells carried between 1 and 3 viral genomes (vg) (Fig. 1e and Extended Data Fig. 1b). In addition, western blot demonstrated that proteins levels in all three genotypes fell within the same range and were not significantly different (Fig. 1f). These results, together with the low variability in functional data derived from electrocardiographic (ECG) and patch-clamp recordings (see below), strongly support the reproducibility and validity of the model. As expected from a trafficking-deficient mutant that is retained at the Golgi apparatus^10^, biochemical analysis using an antibody against the extracellular loop of the channel in non-permeabilized cells showed less Kir2.1^Δ314-315^ protein transported to the plasma membrane compared to mice transduced with the WT isoform (Fig. 1g), confirming a trafficking defect in the heart in vivo.

### The ATS1 mouse recapitulates the patientʼs ECG phenotype

**Fig. 2:**
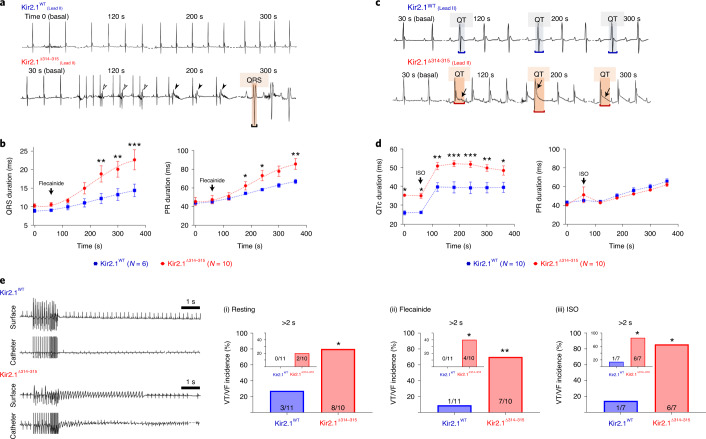
The ATS1 mouse replicates the patientʼs electrical phenotype. **a**,**c**, Representative ECG recordings in Kir2.1^WT^ (top) and Kir2.1^∆314-315^ (bottom) anesthetized mice showing the different ECG pattern in each group at different timepoints after a single dose of flecainide (40 mg kg^−1^) and isoproterenol (isoprenaline, ISO, 5 mg kg^−1^), respectively. **b**, Quantification of temporal changes in QRS and PR interval durations before and after flecainide. Every value represents ten averaged intervals of ten consecutive beats. Data are expressed as mean ± s.e.m. Statistical analyses were conducted using two-way ANOVA, followed by Tukey’s multiple comparisons (QRS at 240 seconds, *P* = 0.0039; 300 seconds, *P* = 0.0027; 360 seconds, *P* = 0.0002 / PR at 240 seconds, *P* = 0.0491; 300 seconds, *P* = 0.0502; 360 seconds, *P* = 0.0107). **d**, Corrected QT and PR intervals before and after ISO dose. Each value represents ten averaged intervals from ten consecutive beats. Data are expressed as mean ± s.e.m. Statistical analyses were conducted using two-way ANOVA, followed by Tukey’s multiple comparisons (QTc at 0 seconds, *P* = 0.0133; 60 seconds, *P* = 0.0203; 120 seconds and 180 seconds, *P* = 0.0025 and *P* = 0.0005; 240 seconds, *P* = 0.0005; 300 seconds, *P* = 0.0048; 360 seconds, *P* = 0.0190). **e**, Representative lead-II ECG traces and corresponding intracardiac recordings from anesthetized Kir2.1^WT^ and Kir2.1^∆314-315^ mice. Graphs show the incidence of VT/VF in basal conditions (i; *P* = 0.0300), after flecainide (ii; *P* = 0.0075) and after ISO (iii; *P* = 0.0291). Animals that had at least one AF episode ≥2 seconds after burst pacing or S1-S2 stimulation are represented as graph insets. A period of polymorphic VT is shown (*P* = 0.2143, *P* = 0.0351 and *P* = 0.0291, for basal, flecainide and isoproterenol, respectively). Each value is represented as mean ± s.e.m. Statistical analyses were conducted using two-tailed *t*-test and Fisher’s exact test. *P < 0.05; **P < 0.01; ***P < 0.001. Source data

**Extended Data Fig. 2:**
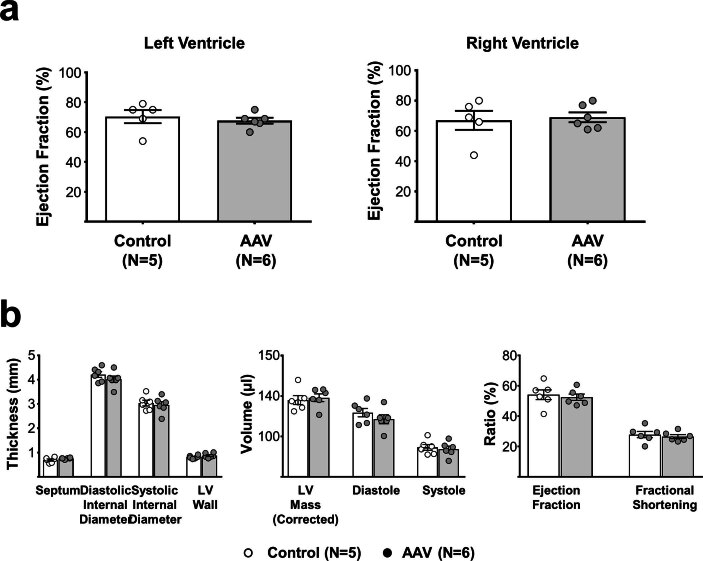
Kir2.1^Δ314-315^ expression does not modify cardiac function. **a**, Quantification of left and right ventricular ejection fraction (EF), by magnetic resonance imaging (MRI) in anesthetized control (WT) and AAV-transduced mice (AAV). **b**, Quantification of septal and left ventricular thickness during systole and diastole (left), Left ventricular volumes (middle), ejection fraction, and fractional shortening (right) by cardiac echocardiography in control (WT) and anesthetized AAV-transduced mice. * = p < 0.05. Each value is represented as the mean ± SEM. Source data

The resting ECG revealed that Kir2.1^Δ314-315^ mice suffered sinus arrhythmia and frequent premature ventricular contractions (PVCs) in the form of bigeminy (Fig. 2a). These electrical abnormalities were independent of any structural or functional defect, assessed by cardiac magnetic resonance (CMR) and ECG imaging in AAV-injected animals compared to non-infected mice (Extended Data Fig. 2). ECG analysis also revealed significantly longer corrected QT interval (QTc) in Kir2.1^Δ314-315^ mice compared to Kir2.1^WT^ mice, whereas the QRS and PR intervals were similar in both groups (Fig. 2a–b; time 0). To unmask and further characterize the electrical phenotype of ATS1 mice, we conducted a flecainide challenge experiment. Administration of a single intraperitoneal (i.p.) dose of the drug led to progressive prolongation in both QRS and PR intervals (Fig. 2b). After flecainide administration, the QRS duration in the Kir2.1^Δ314-315^ mice was more than twice the Kir2.1^WT^ mice, as would be expected by impaired trafficking of the Kir2.1–Na_V_1.5 channelosome produced by the 314-315 internal deletion on Kir2.1 protein^10^. Furthermore, Kir2.1^Δ314-315^ mice showed increased arrhythmogenesis in response to isoproterenol (ISO) administration, with obvious repolarization abnormalities, occasional U-waves (black arrows in Fig. 2c), ventricular extrasystoles and a rapid increase in the QTc interval, without significant changes in the PR interval (Fig. 2d). Kir2.1^Δ314-315^ animals were also more vulnerable than control animals to atrial fibrillation (AF) and ventricular tachycardia/ventricular fibrillation (VT/VF) induced by endocardial burst pacing or the S1-S2 protocol (Fig. 2e). Arrhythmia vulnerability was already present in basal conditions (i) but was especially evident after flecainide (ii) or ISO (iii) administration. In some cases, VT was polymorphic and converted to VF (Fig. 2e). Altogether, the foregoing data demonstrate that arrhythmias in the AAV-mediated ATS1 mouse model are a consequence, in part, of reduced cardiac excitability and conduction, both of which are aggravated by stress.

### Flecainide leads to reentrant and multifocal arrhythmias

**Fig. 3:**
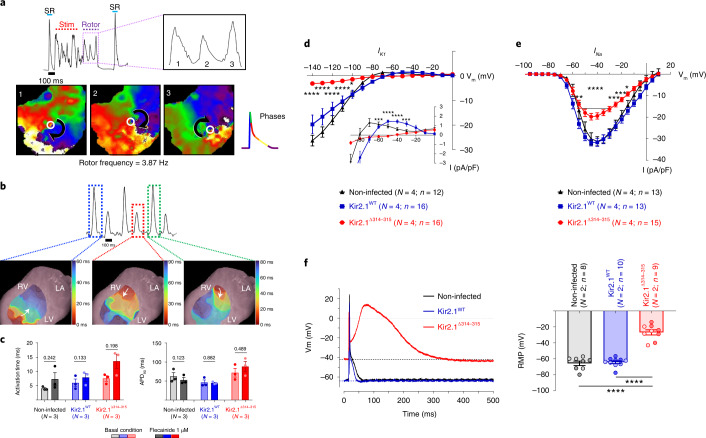
Cardiac electrophysiological defects in the ATS1 model. **a**–**c**, Reentrant and multifocal arrhythmias in ATS1 mouse hearts. **a**, Top, representative single camera pixel recording from an optical mapping experiment in an ATS1 mouse heart in the presence of 1 µM flecainide. Data show arrhythmic behavior induced by a brief tachypacing train (Stim). Three spontaneous APs are observed upon termination of the train (inset). Bottom, Phase maps reveal that those APs were generated by three sequential rotations of an unstable rotor. In each map, the center of rotation (singularity point) is indicated by a white circle; rotor direction is clockwise as indicated by the curved black arrows. **b**, Top, Single pixel recording shows AP amplitude alternans in a different ATS1 mouse heart perfused with 1 µM flecainide. Bottom, 4-ms color isochrone maps show distinctive epicardial breakthrough origin and wave propagation direction for each AP, revealing the multifocal origin of the arrhythmia. **c**, Quantification of epicardial activation time and APD (at 90% of repolarization) during sinus rhythm for non-infected, Kir2.1^WT^ and Kir2.1^∆314-315^ mouse hearts in the presence and absence of 1 µM flecainide. Each value is represented as mean ± s.e.m. One-way ANOVA test among basal conditions groups and two-tailed paired *t*-test between conditions. **d**–**f**, Electrophysiological defects in ATS1 mouse cardiomyocytes. **d**,**e**, I–V relationships of inward rectifying potassium current *I*_*K1*_ (**d**; *P* = 0.0112 at −100 mV; *P* = 0.0001 at −60 mV; and *P* = 0.0004 at −30 mV) and inward sodium current *I*_*Na*_ (**e**; *P* = 0.0030 at −55 mV; *P* = 0.0002 at −25 mV; *P* = 0.0003 at −20 mV; and *P* = 0.0203 at −15 mV) densities in Kir2.1^∆314-315^ compared to WT ventricular cardiomyocytes. Statistical analyses were conducted using two-way ANOVA, followed by Tukey’s multiple comparisons. **f**, Representative AP recordings obtained by current-clamping show depolarized resting membrane potential, reduced excitability and prolonged AP duration in Kir2.1^∆314-315^ versus Kir2.1^WT^ cardiomyocytes. Graph shows the quantification of RMP. Different colors in the same group identify cells coming from one animal. Statistical analyses were conducted using two-level hierarchical one-way ANOVA analysis, followed by Bonferroni’s post test. Each value is represented as mean ± s.e.m. *P < 0.05; **P < 0.01; ***P < 0.001; ****P < 0.0001. Source data

**Extended Data Fig. 3:**
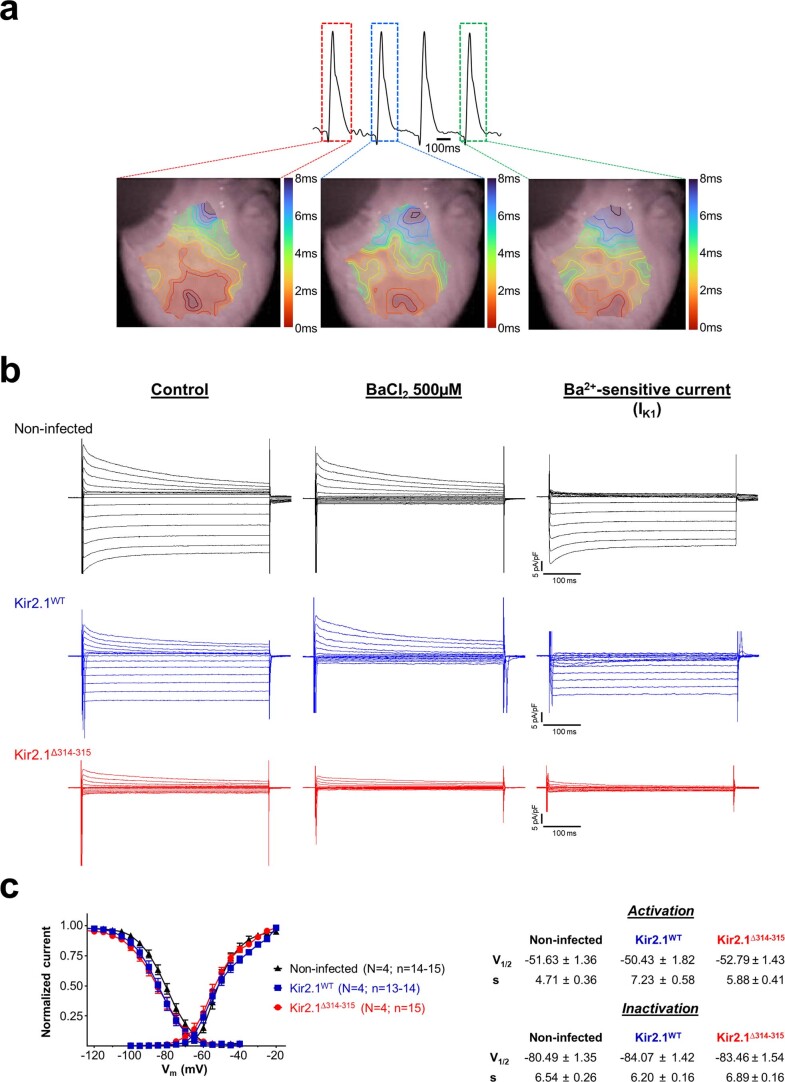
Electrical properties of non-infected, WT and ATS1 mice. **a**, Optical mapping in hearts from non-infected control show normal and fast propagation. Representative single-pixel action potential recordings in a heart of a non-infected control mouse after 1 µM flecainide administration. The three corresponding wave propagation maps at bottom (isochrones are plotted every 0.5 ms), show sequential beat-to-beat epicardial breakthrough patterns emerging from the right ventricular apex (red) and propagating rapidly upward toward the basis, indicative of normal wave propagation during sinus rhythm. **b**, I_K1_ is significantly decreased after Kir2.1^∆314-315^ expression. Representative I_K1_ density traces in control conditions (*left*), after barium perfusion (*center*) and after subtraction of the barium-sensitive current (*right*) from non-infected control (*top*), Kir2.1^WT^ (middle) and Kir2.1^∆314-315^ (bottom) isolated cardiomyocytes. **c**, Cardiac expression of AAV-Kir2.1^Δ314-315^ does not modify *I*_*Na*_ voltage-dependence of activation or inactivation. *Left*, Na channel steady-state activation and inactivation curves for Non-infected, AAV-Kir2.1^WT^ and AAV-Kir2.1^∆314-315^ cardiomyocytes plotted according to the protocol detailed in the Methods section. Currents were plotted as a fraction of peak current and fitted according to a Boltzmann function. *Right*, table shows the activation and inactivation parameters for all experimental groups (Activation V_1/2_: *P* = 0.8790, Non-infected vs Kir2.1^WT^; *P* = 0.8516, n-infected vs Kir2.1^∆314-315^; and, *P* = 0.5371, Kir2.1^WT^ vs Kir2.1^∆314-315^. Activation slope (s): *P* = 0.0030, Non-infected vs Kir2.1^WT^; *P* = 0.1514, n-infected vs Kir2.1^∆314-315^; and, *P* = 0.1037, Kir2.1^WT^ vs Kir2.1^∆314-315^. Inactivation V_1/2_: *P* = 0.3521, Non-infected vs Kir2.1^WT^; *P* = 0.4322, n-infected vs Kir2.1^∆314-315^; and, *P* = 0.9555, Kir2.1^WT^ vs Kir2.1^∆314-315^. Inactivation slope (s): *P* = 0.8386, Non-infected vs Kir2.1^WT^; *P* = 0.0410, n-infected vs Kir2.1^∆314-315^; and, *P* = 0.0186, Kir2.1^WT^ vs Kir2.1^∆314-315^). Each value is represented as the mean ± SEM. Statistical analyses were conducted using One-way ANOVA test followed by Tukey’s multiple comparisons posttest. Source data

To investigate flecainide-induced arrhythmogenesis, we carried out optical mapping experiments in isolated mouse hearts (Fig. 3). Color phase mapping revealed that, upon 1 µM flecainide administration, a burst of tachypacing was followed by non-sustained reentrant activity (rotors) with three complete rotations, followed by spontaneous termination (Fig. 3a). Clearly, these highly abnormal patterns of wave propagation contrast with the normal epicardial breakthrough patterns demonstrated in non-infected control mice during sinus rhythm (Extended Data Fig. 3a). As such, flecainide exacerbates the already abnormal conduction in the ATS1 mouse heart, leading to reentry and polymorphic VT. In addition, action potential (AP) amplitude alternans appeared in the mutant mouse hearts as multifocal epicardial breakthrough patterns (Fig. 3b), distinct from the normal patterns seen in the control experiments (Extended Data Fig. 3a). Importantly, similar multifocal epicardial patterns have been demonstrated in CPVT mice because of triggered discharges in subendocardial Purkinje fibers, which gave rise to bidirectional tachycardia^18,19^. Concomitantly with the various arrhythmic patterns, optical mapping revealed abnormally prolonged electrical activation times and action potential duration (APD) upon flecainide administration in the ATS1 mice (Fig. 3c).

### Impaired Kir2.1–Na_V_1.5 channelosome function in ATS1 mice

In accordance with previous in vitro reports^10^, patch-clamp experiments demonstrated that cardiomyocytes from Kir2.1^Δ314–315^ mice had reduced I_K1_ compared to WT (Fig. 3d and Extended Data Fig. 3b). Moreover, as illustrated in Fig. 3e, cardiomyocytes from AAV-Kir2.1^Δ314–315^ mice exhibited a ~35% reduction in sodium current (I_Na_) density compared to cardiomyocytes from mice transduced with AAV-Kir2.1^WT^. However, such differences were not due to differences in their voltage-dependence of activation or inactivation (Extended Data Fig. 3c). Our data predicted that, in current-clamp assays, the resting membrane potential (RMP) of Kir2.1^Δ314–315^ should be more depolarized than controls. Indeed, this was the case as only one of nine (~11%) cells were able to generate evoked APs but with substantially longer duration than WT (Fig. 3f).

### Kir2.1 localizes in two microdomains in cardiomyocytes

**Fig. 4:**
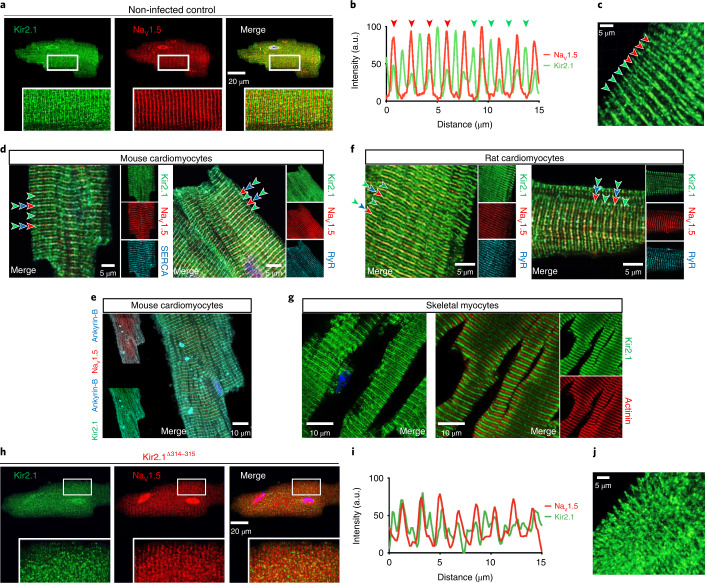
Two separate Kir2.1 bands in cardiac and skeletal myocytes. **a**,**b**, Confocal images (**a**) and fluorescence intensity profiles (**b**) of isolated cardiomyocytes from control mice show the double Kir2.1 expression pattern in comparison with Na_V_1.5 channels. Arrows point to Na_V_1.5 channels in red and predominant Kir2.1 channels in green. Note the clearly discernible Kir2.1 expression pattern occurring at regular ~0.9-µm intervals, whereas Na_V_1.5 appears at 1.8-µm intervals. These images (**a** and **b**) are representative of 70 cells from 12 animals analyzed. **c**, Tri-dimensional reconstruction of Kir2.1 staining of bands close to Na_V_1.5 channels (red arrows) and those of separate Kir2.1 channels (green arrows). **d**,**f**, Confocal mouse (**d**) and rat (**f**) cardiomyocyte images showing the location of Kir2.1 channels together with SERCA (left) and RyR2 (right). These images (**d** and **f**) are representative of 15 cells from three animals analyzed in each group. **e**, Confocal images of isolated mouse cardiomyocytes from control mice showing the Kir2.1 expression pattern in comparison with Na_V_1.5 and ankyrin-B. This image is representative of 14 cells from three animals analyzed. **g**, Confocal images of mouse skeletal muscle slices showing the double Kir2.1 localization pattern—Kir2.1 alone (left) and Kir2.1 plus actinin (right). These images are representative of seven slices from three animals in each group. **h**,**i**, Representative confocal images (**h**) and fluorescence intensity profiles (**i**) of isolated cardiomyocytes from Kir2.1^∆314-315^ mice showing disrupted expression patterns of Kir2.1 and Na_V_1.5 channels. These images are representative of 27 cells from six animals. **j**, Tri-dimensional reconstitution of Kir2.1 staining showing the disorganization of the Kir2.1 expression pattern of a Kir2.1^∆314-315^ cardiomyocyte. a.u., arbitrary units.

**Extended Data Fig. 4:**
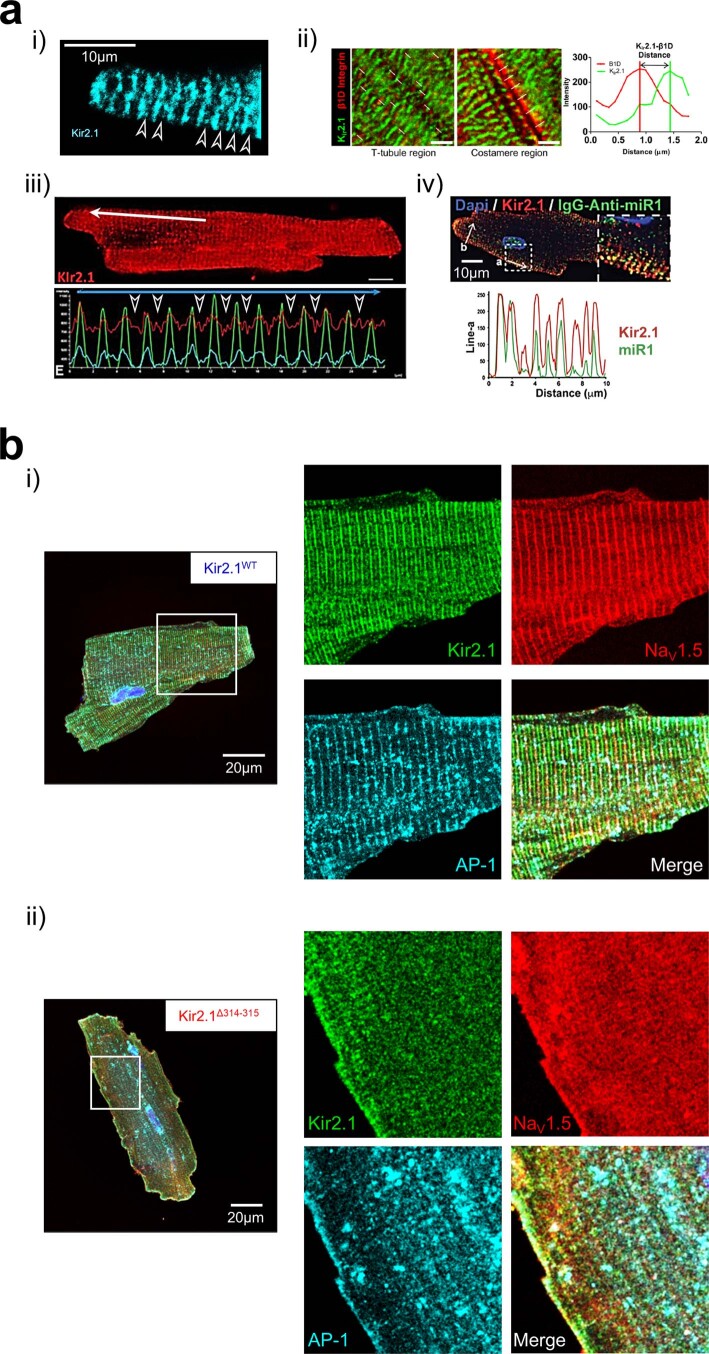
The extra Kir2.1 band was there but not seen previously. **a**, Presence of Kir2.1 at the SR is suggested in previously published immunostaining images, as follows: i) Inter-tubular Kir2.1 staining (white arrowheads) in isolated rat cardiomyocytes (from Ponce-Balbuena et al. 2018^1^); ii) Magnified images of t-tubule (*left*) and costamere (middle) regions stained for Kir2.1 with ß1D integrin as t-tubule marker. Representative green and red fluorescence intensity profiles along one of the white lines shown on left and middle. Note the presence of Kir2.1 bands intercalated with those of ß1D (from Sengupta et al. in 2019);^2^ iii) Inter-tubular Kir2.1 staining (white arrowheads) in isolated rat cardiomyocytes (from Park et al. in 2020^3^); iv) Immunostaining (*top*) and traces of fluorescence intensity spatial profiles (*bottom*) of Kir2.1 and miR1 through ‘a’ arrow-line in immunostaining. *Note the presence of one line every* ≈ *1* *µm* (from Yang et al. in 2021)^4^. All Figures reproduced by permission of the respective authors and journals. **b**, Mislocalization of Adaptin in Kir2.1^Δ314-315^ cardiomyocytes likely contributes to Na_V_1.5 mislocalization. Confocal images show the expression pattern of Adaptin (AP1), Na_V_1.5 and Kir2.1 in Kir2.1^WT^ (i) and Kir2.1^Δ314-315^ (ii) cardiomyocytes. These images are representative of 24 cells from 3 animals analyzed.

Kir2.1 co-localizes with Na_V_1.5 forming channelosomes with various adaptor and scaffolding proteins at lateral membranes, t-tubules and intercalated discs^8,10^. Representative confocal images of ventricular cardiomyocytes from normal non-infected mice showed co-localization of Kir2.1 and Na_V_1.5 (Fig. 4a), where RyR type-2 (RyR2) and SERCA also co-localized at the Z line (Fig. 4d). These confocal images also revealed that Kir2.1 localized in a defined structure running parallel to the t-tubule at ~0.9 µm (Fig. 4b,c), halfway between two Z lines. The location of Kir2.1 at this microdomain corresponded to the M line where Kir2.1 co-localized with ankyrin-B (Fig. 4e). Furthermore, although a double Kir2.1 band pattern has not been specifically reported previously, it is clearly visible in several published illustrations from different laboratories^10,12,20,21^ (Extended Data Fig. 4a). To discard the possibility that the double Kir2.1 band pattern was mouse specific, we analyzed Kir2.1 immunolocalization in rat cardiomyocytes, confirming that Kir2.1 is localized in two distinct microdomains in both species (Fig. 4f). More importantly, we also proved the presence of the double-band pattern of Kir2.1 in normal mouse skeletal muscle tissue sections (Fig. 4g), suggesting a potentially important role of both pools of Kir2.1 protein in different types of muscle cells.

Trafficking of both Kir2.1 and Na_V_1.5 to their membrane microdomains depends on their incorporation into clathrin-coated vesicles at the trans-Golgi network marked by interaction with the AP1 (adaptor protein complex 1) ϒ-adaptin subunit in both mice and rat^10,16^. As shown in Fig. 4h–j, in contrast with the normal non-infected group, Kir2.1^Δ314-315^ expression disrupted the well-demarcated organization of both the Kir2.1–Na_V_1.5 channelosome band and the parallel band where Kir2.1 is expressed alone. On the other hand, as illustrated in Extended Data Fig. 4b, in WT mouse cardiomyocytes, AP1 ϒ-adaptin displayed a clear co-localization with the Na_V_1.5 channelosome and little or no staining near the M line. However, the pattern was less organized in ATS1 mouse cardiomyocytes, showing a patchy distribution at several locations, likely due to the lack of a recognition site for interaction with AP1 in the Kir2.1^Δ314-315^ protein, a key mediator of Kir2.1 and Na_V_1.5 trafficking and membrane targeting^10,16^.

### A Kir2.1 protein subpopulation localizes at the SR

**Fig. 5:**
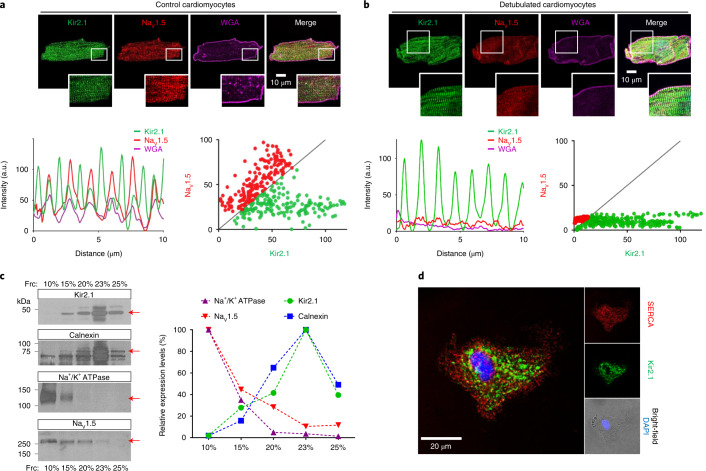
The additional Kir2.1 band is at the SR membrane. **a**, Top, confocal images of Kir2.1, Na_V_1.5 and cardiomyocyte membrane (WGA). Bottom, fluorescence profiles (right) and Na_V_1.5–Kir2.1 fluorescence correlation in isolated control (left). **b**, Top, confocal images of Kir2.1, NaV1.5 and cardiomyocyte membrane (WGA) after formamide-mediated detubulation of cardiomyocytes reveals the intracellular Kir2.1 band alone. Bottom, Fluorescence profiles show absence of Nav1.5 staining at the t-tubules (right), with absence of Na_V_1.5–Kir2.1 fluorescence correlation (left). **c**, Left, Kir2.1, calnexin, Na^+^/K^+^ ATPase and Na_V_1.5 analysis by western blot after ventricle membrane fractionation in 10–25% of iodixanol. Right, Graph shows quantification of protein analysis shown in left. **d**, Representative confocal image (*n* = 10 from three animals) of Kir2.1 and SERCA in the SR network connected with the envelope of an isolated nucleus. a.u., arbitrary units. Source data

To discriminate whether the previously unrecognized Kir2.1 protein band is in the sarcolemma or in an intracellular membrane compartment, we performed formamide-mediated detubulation assays (Fig. 5a,b). Confocal images of cardiomyocytes from normal non-infected mice stained to detect both Kir2.1 and Na_V_1.5 showed two clearly separate, alternating Kir2.1 protein localizations, one isolated and the other co-localizing with Na_V_1.5 channels (Fig. 5a). However, upon formamide-mediated detubulation, the sarcolemma stained with wheat germ agglutinin (WGA) reflected t-tubular system disruption, and Kir2.1 and Na_V_1.5 staining was clearly obliterated (Fig. 5b). In contrast, the unique Kir2.1 second structure associated with the M line remained intact despite detubulation, indicating its location in an intracellular compartment. Concordant with the data shown above and those described previously^8,10,15^, membrane fractionation using iodixanol-mediated density gradient showed that Na_V_1.5 channels segregated into two different populations, one Kir2.1-independent at the 10% fraction and the other Kir2.1-dependent at the 15% and 20% fractions (Fig. 5c). On the other hand, the largest proportion of Kir2.1 channels were Na_V_1.5-independent and isolated along with the specific SR protein calnexin (23% fraction), supporting the localization of Kir2.1 at the SR cellular domain (Figs. 4 and 5a,b). Furthermore, direct visualization of SR vesicles isolated together with cell nuclei revealed that Kir2.1 protein co-localized with SERCA, the major SR calcium transporter (Fig. 5d). Altogether, the data demonstrate that the M-line-associated Kir2.1 band is located at an SR microdomain.

### SR vesicular membranes display a rectifying SR K^+^ current

**Fig. 6:**
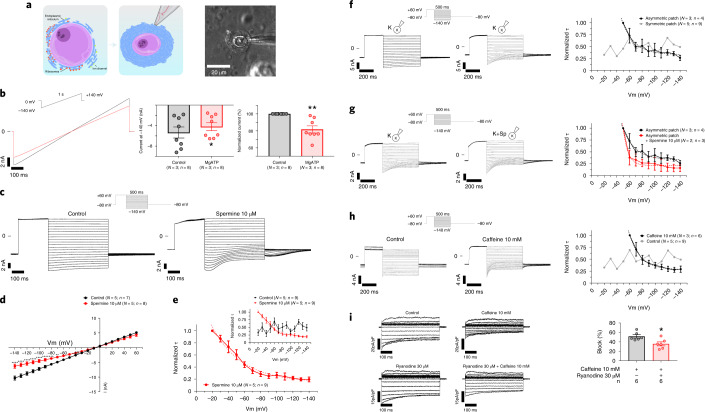
SR Kir2.1 channels are functional and show rectification. **a**. Schematic representations (left) and phase contrast micrograph (right) of an isolated SR vesicle attached to the nucleus of a cardiomyocyte from normal non-infected mice in a patch-clamp experiment. This scheme was performed and considered representative after 30 perinuclear vesicles recorded from 14 animals. **b**, Representative patch-clamp recordings obtained by applying the voltage ramp protocol shown on top in the absence (black) and presence of 2 mM MgATP. Graphs show the magnitude currents in absolute values (left; *P* = 0.0267) and normalized to each control experiment (right, *P* = 0.0032). **c**, Representative patch-clamp recordings obtained by applying the voltage protocol shown on top in the absence (left) and presence (right) of spermine 10 µM. **d**, I–V relationships constructed at the end of the test pulses in **b** for control (black) and spermine (red). **e**, Time constant (τ) of block by spermine 10 µM (obtained from **c**) saturates at negative membrane potentials. Inset shows the same spermine data (red) compared to control (black). τ values were estimated using monoexponential fits. **f**–**h**, Left, representative patch-clamp experiment recordings using the voltage protocol shown on top. Right, time constant (τ) of block shown in left panels. **f**, Effect of residual endogenous spermine present in the SR vesicles under voltage-clamp conditions with symmetrical versus asymmetrical K^+^ concentration. **g**, Effect of spermine 10 µM after asymmetrical patch-clamping. **h**, Effect of caffeine 10 mM. τ values were estimated using monoexponential fits. **i**, Left, representative recordings from HEK293 cells transfected with Kir2.1 channels before and after perfusion with caffeine 10 mM. Right, quantification of caffeine-induced block at −120mV (*P* = 0.0175). Each value is represented as mean ± s.e.m. Statistical analyses were conducted using two-tailed *t*-test and two-way ANOVA test. *P < 0.05; **P < 0.01; ***P < 0.001; ****P < 0.0001. Source data

**Extended Data Fig. 5:**
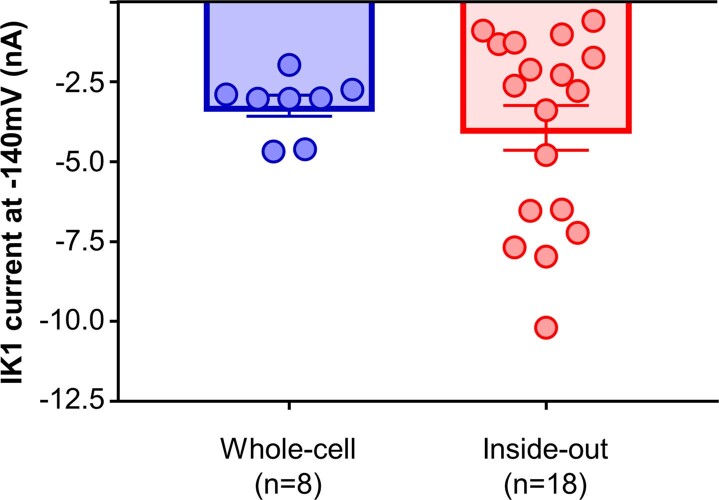
Current magnitude in SR vesicles differs from whole cell. Analysis of I_K1_ magnitude in different experimental conditions. Graphs show the current magnitude at −140mV in whole cell and inside-out patch. Note that in the inside-out patch the magnitude of the currents is much higher than expected in comparison with the current recorded in the whole-cell configuration. This phenomena is likely due to the differences in potassium concentrations comparing whole-cell (5.4 mM in the bath solution and 138 mM in the pipette) and whole-vesicle currents (150 mM at both sides). Each value is represented as the mean ± SEM. Statistical analysis is not applicable. Source data

To assess whether the SR Kir2.1 channels were functional, we carried out patch-clamp experiments in the SR vesicles around segregated nuclei from normal non-infected cardiomyocytes (Fig. 6a). Voltage ramps from −140 mV to +140 mV elicited currents whose large magnitude likely depended on the quality of the seals formed between the vesicles and the pipette, influenced by the SR vesicle membrane composition. In addition, the symmetric 150 mM K^+^ solution also contributed to the large magnitude of the currents, as revealed by inside-out patch experiments carried out in HEK293 cells (Extended Data Fig. 5).

**Extended Data Fig. 6:**
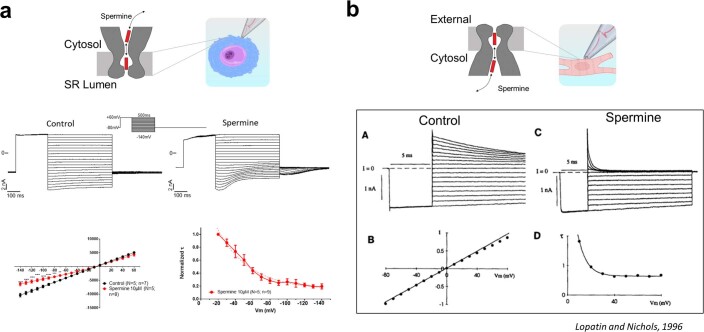
Direction of rectification depends on channel orientation. **a**, *Top*, Schematic diagram of expected orientation of SR Kir channel with respect to SR lumen and cytosol in a nuclear membrane experiment. *Middle*, Representative recordings of SR Kir2.1 currents in control and in the presence of spermine. *Bottom*, Current voltage relationship and normalized SR Kir2.1 current (same data as in Fig. 6c-e**)**. Each value is represented as mean ± SEM. Statistical analyses were conducted using Two-way ANOVA test. **, p < 0.01; ***, p < 0.001; ****, p < 0.0001. **b**, *Top*, Schematic diagram of expected orientation of sarcolemmal Kir channel as reported by Nichols and Lopatin (1996)^5^. *Middle*, Representative recordings of sarcolemmal Kir2.1 currents in control and in the presence of spermine. *Bottom*, Current voltage relationship and normalized sarcolemmal Kir2.1 current. Note that differences between data in **a** and **b** are likely related to differences in channel orientation within each lipid bilayer. Figure reproduced with permission of the journal.

**Extended Data Fig. 7:**
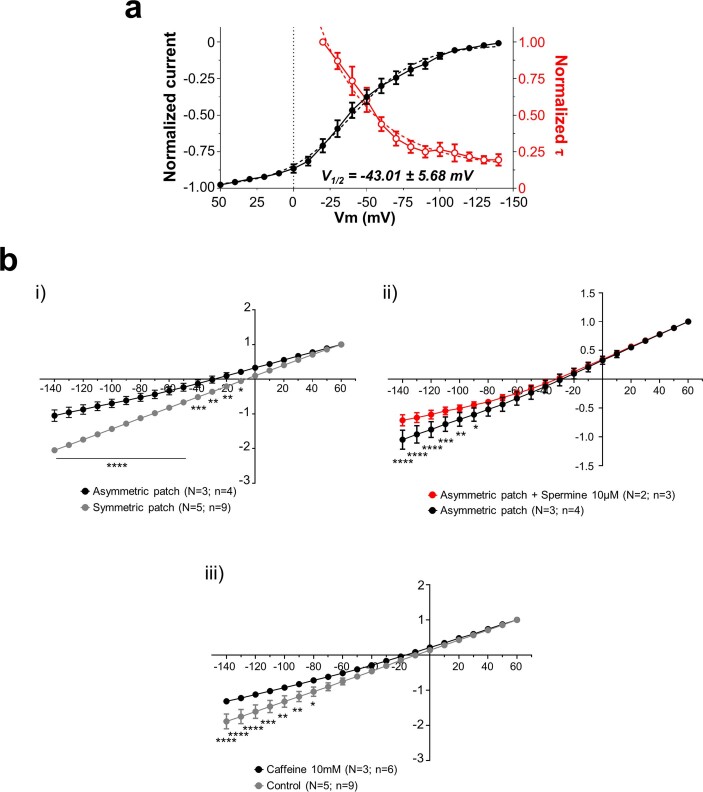
Rectifying properties of Kir2.1 channels in SR vesicles. **a**, Voltage-dependent effect of spermine as seen in the tail potassium currents (in black). Tail current increases as the time constant (τ) of block by spermine decreases (in red). Each value is represented as mean ± SEM. Statistical analysis is not applicable. **b**, Normalized current-voltage (I-V) relationships constructed at the end of the test pulse. i) Effect of residual endogenous spermine present in the SR vesicles under voltage-clamp conditions with symmetrical versus asymmetrical K + concentration (*P* < 0.0001 for voltages ≤ −50mV; *P* = 0.0002, for −40mV; *P* = 0.0013, for −30mV; *P* = 0.0087, for −20mV; *P* = 0.0438, for −10mV) ii) Effect of spermine 10 µM after asymmetrical patch-clamping (*P* < 0.0001 for voltages ≤ −120mV; *P* = 0.0004, for −110mV; *P* = 0.0055, for −100mV; *P* = 0.0232, for −90mV). iii) Effect of caffeine 10 mM (*P* < 0.0001 for voltages ≤ −120mV; *P* = 0.0003, for −110mV; *P* = 0.0017, for −100mV; *P* = 0.0080, for −90mV; *P* = 0.0240, for −80mV). Each value is represented as mean ± SEM. Statistical analyses were conducted using Two-way ANOVA test. *, p < 0.05; ***p < 0.01 ***, p < 0.001; ****, p < 0.0001. Source data

Voltage-clamping of the SR vesicles revealed magnesium-sensitive (Fig. 6b) and spermine-sensitive (Fig. 6c–e) potassium currents with similar properties but opposite polarity to I_K1_ (refs. ^22–24^) (Fig. 6c–e and Extended Data Fig. 6), whose endogenous spermine-induced rectification was voltage-dependent (Extended Data Fig. 7a) and potentiated by exposure to asymmetrical potassium concentrations (Fig. 6f,g and Extended Data Fig. 7b)^25^. Together, these data strongly suggest that SR Kir2.1 channels are functional and oriented to provide a K^+^ current that rectifies (decreases) in the direction of the SR lumen (hereafter termed ‘rectifying SR K^+^ current’).

The location and orientation of the rectifying SR Kir2.1 channels leads us to surmise that they contribute a countercurrent in the regulation of calcium movements across the SR, as has been suggested for K^+^ for many years^26–28^. Thus, we analyzed the effect of caffeine perfusion, a strong RyR agonist, on the rectifying SR K^+^ current of the nuclear vesicles, which produced a strong spermine-like effect (Fig. 6h and Extended Data Fig. 7b). To determine whether caffeine acts directly on the Kir2.1 channel, or whether the effect is secondary to Ca^2+^ dynamic activation, we conducted similar experiments in HEK293 cells, in the absence and presence of 30 µM ryanodine. The results demonstrated a direct effect of caffeine on the rectifying SR K^+^ current (Fig. 6i). Altogether, these data support the hypothesis that caffeine-sensitive SR Kir2.1 channels have an important role in the control of the intracellular calcium dynamics and that caffeine promotes Ca^2+^ efflux by acting simultaneously both as an agonist to RyR and as an antagonist to the rectifying SR K^+^ countercurrent.

### SR Kir2.1 channels modulate intracellular Ca^2+^ dynamics

**Fig. 7:**
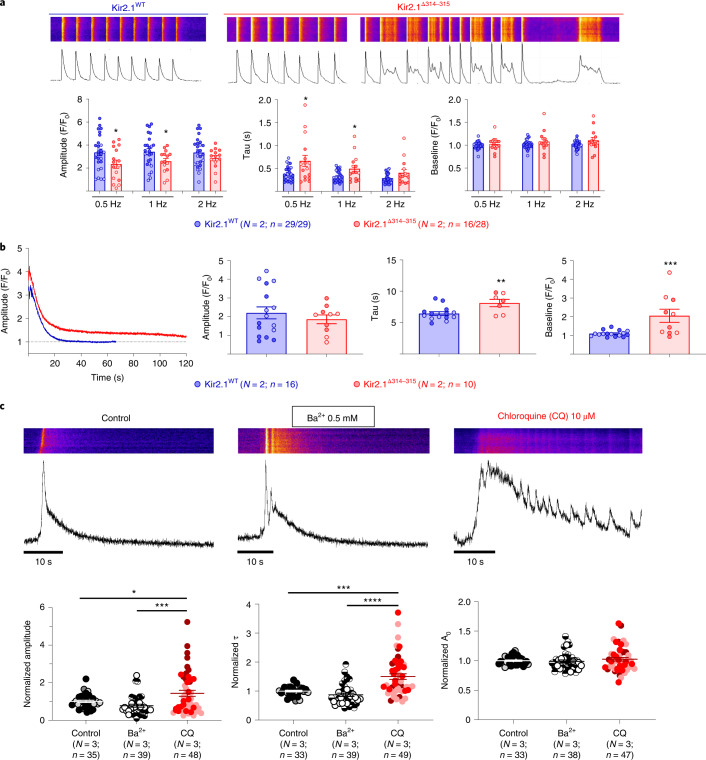
Abnormal calcium dynamics in ATS1 mouse cardiomyocytes. **a**, Analysis of calcium dynamics in response to stimulation at 0.5 Hz, 1 Hz and 2 Hz. Graphs show amplitude (*P* = 0.0408 at 0.5 Hz; *P* = 0.0420 at 1 Hz; and *P* = 0.2199 at 2 Hz), tau (decay kinetics; *P* = 0.0102 at 0.5 Hz; *P* = 0.0194 at 1 Hz; and *P* = 0.0628 at 2 Hz) and baseline (*P* = 0.3978 at 0.5 Hz; *P* = 0.1892 at 1 Hz; and *P* = 0.0520 at 2 Hz) of each Ca^2+^ transient. Note that, in the representative Kir2.1^∆314-315^, cardiomyocyte e–c coupling shows multiple abnormal spontaneous calcium release events during both systole and diastole, which are absent in the Kir2.1^WT^ cardiomyocyte. **b**, Analysis of Ca^2+^ transient decay after perfusion of caffeine 10 mM during 20 seconds (*P* = 0.8725, *P* = 0.0069 and *P* = 0.0006 for the amplitude, tau and baseline, respectively). **c**, Representative fluorescence profiles (top) of caffeine-induced calcium release in control conditions and in the presence of 0.5 mM Ba^2+^ or 10 µM chloroquine (CQ) in isolated cardiomyocytes from normal non-infected control mice. Graphs (bottom) show the parameters obtained after monoexponential fit to calcium reuptake and release, respectively (Amplitude: *P* = 0.3056, control versus Ba^2+^; *P* = 0.0113, control versus CQ; and *P* = 0.0001, Ba^2+^ versus CQ. Tau: *P* = 0.8002, control versus Ba^2+^; *P* = 0.00003, control versus CQ; and *P* < 0.0001, Ba^2+^ versus CQ. Baseline: *P* = 1.0000, control versus Ba^2+^; *P* = 1.0000, control versus CQ; and *P* = 0.9091, Ba^2+^ versus CQ*)*. Each value is represented as the mean ± s.e.m. Different colors in the same group identify cells coming from one animal. Statistical analyses were conducted using two-level hierarchical *t*-test (**a**,**b**) and one-way ANOVA (**c**) analysis followed by Bonferroni’s post test. *P < 0.05; ***P < 0.001; ****P < 0.0001. Source data

**Extended Data Fig. 8:**
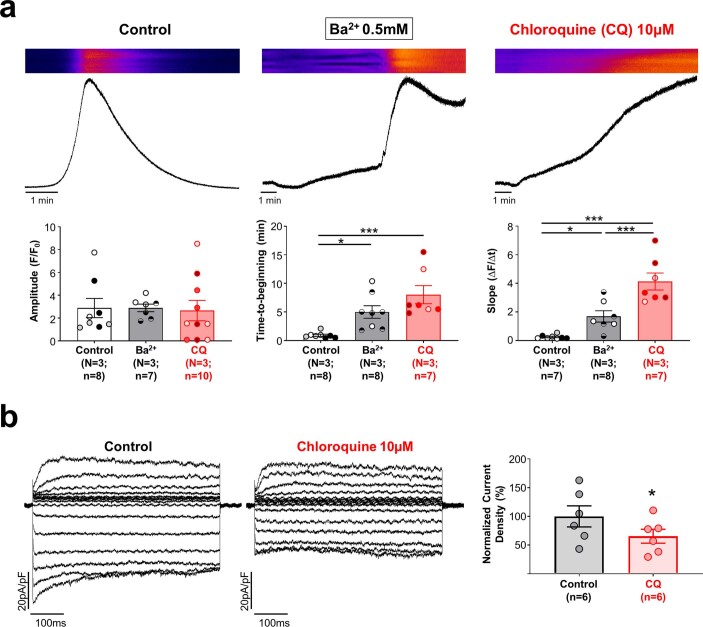
Chloroquine effects on intracellular Ca^2+^ dynamics in skeletal myocytes. **a**, Representative fluorescence profiles (top) of caffeine-induce calcium release in control conditions and in the presence of Ba^2+^ 0.5 mM or chloroquine 10 µM in isolated skeletal muscle cells. Graphs (bottom) show the parameters obtained after Boltzman fit (Amplitude: *P* = 1.000, Control vs Ba^2+^; *P* = 0.9771, Control vs CQ; and, *P* = 0.9771, Ba^2+^ vs CQ. Tau: *P* = 0.0445, Control vs Ba^2+^; *P* < 0.0001, Control vs CQ; and, *P* = 0.0011, Ba^2+^ vs CQ. Baseline: *P* = 0.0308, Control vs Ba^2+^; *P* = 0.0004, Control vs CQ; and, *P* = 0.1429, Ba^2+^ vs CQ). Different colors in the same group identify cells coming from one animal. Statistical analysis was conducted using two-level hierarchical one-way ANOVA analysis followed by a Bonferroni’s posttest. Each value is represented as the mean ± SEM. *, p < 0.05; ***, p < 0.001. **b**, Representative recordings of HEK cells transfected with Kir2.1 channels before and after perfusion with chloroquine 10 µM (*P* = 0.0282). Graphs show the percentage of block at −120mV. Each value is represented as mean ± SEM. Statistical analyses were conducted using two-tailed paired t-test. *, p < 0.05. Source data

The location and functional characteristics of the previously unidentified rectifying SR K^+^ channels indicate that they likely play a role in the control of intracellular calcium homeostasis. After all, the existence in the SR of potassium and other monovalent channels contributing to the bidirectional SR movement of calcium via RyR2 and SERCA has been suggested for many years^29–32^. Thus, we analyzed the intracellular calcium dynamics in both WT and ATS1 mice (Fig. 7). Cardiomyocytes expressing Kir2.1^Δ314-315^ had an e–c coupling defect in the form of slower calcium transient decay than WT cells after both field stimulation (Fig. 7a) and caffeine administration (Fig. 7b). Consequently, Kir2.1^Δ314-315^ cardiomyocytes showed multiple abnormal spontaneous calcium release events during systole and diastole (Fig. 7a). To establish whether SR Kir2.1 channels have a role in the potassium-mediated Ca^2+^ countercurrent, we measured the kinetics of the caffeine-mediated intracellular Ca^2+^ transient release in isolated cardiac (Fig. 7c) and skeletal (Extended Data Fig. 8a) myocytes in the presence and absence of one of two different inhibitors of I_K1_: Ba^2+^ (0.5 mM; blocks 100% I_K1_), which is a polar ion that blocks Kir2.1 from the external surface of the sarcolemma; and chloroquine 10 µM, which selectively blocks 30% I_K1_ (Extended Data Fig. 8b). Due to its hydrophobicity, chloroquine exerts its ion channel blocking effects through the cytoplasmic side of the channel (Fig. 7). Analysis of the kinetics demonstrated that, unlike Ba^2+^, incubation with chloroquine slowed the calcium dynamics in both cell types. These data strongly support the idea that the chloroquine effect was mediated by blockade of the SR Kir2.1 channels.

**Fig. 8:**
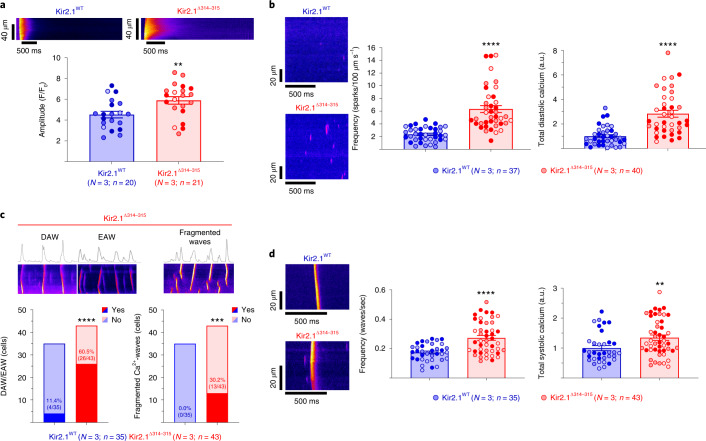
Abnormal Ca^2+^ dynamics in permeabilized ATS1 cardiomyocytes. **a**, Representative fluorescence profiles (top) and analysis of SR load, determined as the amplitude of Ca^2+^ release after perfusion of caffeine 20 mM (*P* = 0.0058). **b**, Left, representative confocal line-scan images of Ca^2+^ sparks (in purple). Analysis of the mean frequency of Ca^2+^ sparks (middle), reported as the average number of events per second in a 100-μm line, and total diastolic calcium release (right), as the total spark Mass (Amplitude×FullWidth×FullDuration) normalized to the area (µm s^−1^) of the line-scan recorded. **c**, Top, representative confocal line-scan images of disturbed Ca^2+^ waves in the form of delayed after Ca^2+^ wave (DAW), early after Ca^2+^ wave (EAW) and fragmented Ca^2+^ waves. Bottom, analysis of the aberrant spontaneous Ca^2+^ release incidence (*P* < 0.0001 and *P* = 0.0003 for DAWs and EAWs, respectively). Statistical analysis was conducted using Fisher’s exact test. **d**, Representative confocal line-scan images of Ca^2+^ waves (left). Analysis of the mean frequency of Ca^2+^ waves (middle) and of total systolic calcium release (right), as the total fluorescence of each line normalized to the mean resting fluorescence (F/F_0_) normalized to the area (µm s^−1^) of the line-scan recorded (*P* < 0.0001 and *P* = 0.0048 for frequency and total systolic Ca^2+^, respectively). Each value is represented as the mean ± s.e.m. Different colors in the same group identify cells coming from one animal. Statistical analyses were conducted using two-level hierarchical *t*-test (**a**,**b**,**d**) analysis, followed by Bonferroni’s post test. *P < 0.05; **P < 0.01; ***P < 0.001; ****P < 0.0001. a.u., arbitrary units. Source data

**Extended Data Fig. 9:**
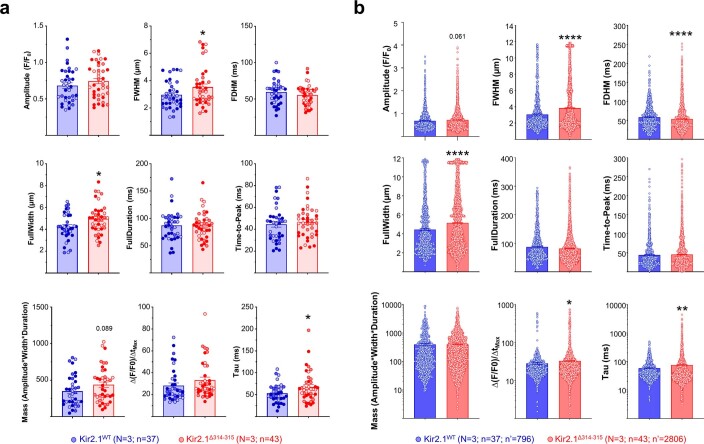
ATS1 cardiomyocytes show altered diastolic Ca^2+^ release. Analysis of all parameters from cells (**a**) or individual sparks (**b**) recorded: Peak Amplitude (F/F_0_; *P* = 0.2256 in a; *P* = 0.0611 in b), Full-Width at Half Maximum (FWHM; μm; *P* = 0.0470 in a; *P* < 0.0001 in b), Full-Duration at Half Maximum (FDHM, ms; *P* = 0.1770 in a; *P* < 0.0001 in b), Full-Width (μm; *P* = 0.0139 in a; *P* < 0.0001 in b), Full-Duration (ms; *P* = 0.9583 in a; *P* = 0.0243 in b), Time-to-Peak (ms; *P* = 0.4010 in a; *P* = 0.4093 in b), Mass (calculated as the peak amplitude multiplied by FullWidth by FullDuration; *P* = 0.1771 in a; *P* = 0.0433 in b), Maximum steepness of spark upstroke [∆(F/F_0_)/∆t_Max_; *P* = 0.0417 in a; *P* = 0.0083 in b], and exponential time constant of the spark decay (Tau, ms; *P* = 0.0873 in a; *P* = 0.3955 in b). Each value is represented as the mean ± SEM. Different colors in the same group identify cells coming from one animal. Statistical analyses were conducted using two- (**a**) and three-level (**b**) hierarchical analysis. *, p < 0.05; **, p < 0.01; ***, p < 0.001. Source data

We then tested whether the arrhythmogenic Ca^2+^ release events also occur in the absence of the plasmalemma. Therefore, we investigated intracellular Ca^2+^ dynamics (SR content, non-propagating Ca^2+^ sparks and Ca^2+^ waves) in permeabilized Kir2.1^WT^ and Kir2.1^Δ314-315^ cardiomyocytes (Fig. 8), as previously described^33,34^. The SR Ca^2+^ content, estimated as the amplitude of the caffeine-induced Ca^2+^ transient, was higher in Kir2.1^Δ314-315^ compared to Kir2.1^WT^ cardiomyocytes (Fig. 8a). In addition, permeabilized Kir2.1^Δ314-315^ cardiomyocytes had more frequent Ca^2+^ diastolic release (≈3-fold) with higher total diastolic calcium release (Fig. 8b) due to alterations in Ca^2+^ spark properties, which were wider and slower than Kir2.1^WT^-expressing cells (Extended Data Fig. 9). Of note, Kir2.1^Δ314-315^ cardiomyocytes also showed a higher incidence of aberrant Ca^2+^ waves in the form of delayed and early after-waves (DAWs and EAWs, respectively; Fig. 8c), consistent as previously described for CPVT mouse models^19,35^. Finally, the frequency of systolic Ca^2+^ release was ≈1.5-fold higher, and the total systolic Ca^2+^ release was also higher in permeabilized Kir2.1^Δ314-315^ than WT cardiomyocytes (Fig. 8d). Altogether, our results provide a clear molecular mechanism for the spontaneous and induced arrhythmias observed in the ATS1 mouse and the phenotypic overlap between ATS1 and CPVT in some patients^5,6^.

## Discussion

Our AAV-mediated cardiac-specific mouse model of ATS1 expressing the trafficking-deficient mutant Kir2.1^Δ314-315^ protein recapitulates the electrophysiological phenotype of patients with ATS1. In this disease model, arrhythmogenicity is due to a dual dysfunction of the mutant Kir2.1 channels: one at the sarcolemma, resulting in reduced excitability and abnormal conduction, and the other at the SR membrane, where the mutant SR Kir2.1 channels directly alter intracellular calcium dynamics.

We demonstrate the existence of two spatially and functionally separate pools of Kir2.1 protein. One population co-localizes with Na_V_1.5 and AP1 near the Z band, and the other co-localizes with ankyrin-B near the M line. We surmise that the localization of the latter population may be due to interaction with some of the proteins involved in the M line complex formed by ankyrin-B, which interacts with the IP3 receptor at the SR^36,37^. Although in vitro experiments had already shown evidence of the formation of Kir2.1–Na_V_1.5 channelosomes at the cellular membrane^8,10^, we are unaware of any previous studies reporting Kir2.1 localization at the M line microdomain. Using a combination of imaging, biochemical and electrophysiological approaches, we demonstrate that the latter pool of Kir2.1 channels is localized with inverted polarity at the SR membrane. These distinct Kir2.1 protein microdomains are present in different species and different muscular cells, which suggests a conserved and generalized function.

A substantial amount of literature since the 1960s has strongly suggested the existence of monovalent ion channels contributing countercurrent to the movements of calcium mediated by RyR2 and SERCA^29–32^. Such countercurrents are necessary to balance the movement of charges across the SR of both skeletal and cardiac muscle^26^, in such a way that the Ca^2+^ equilibrium potential is reached rapidly, preventing any further release^28^. Although calcium movement is accompanied by a voltage change across the SR membrane^27^, the RyR2-mediated SR movement of Ca^2+^ is well-balanced by concomitant monovalent ion countercurrents^27^. To date, the identity of the monovalent channels at the SR had remained controversial^26,28,38,39^. Our calcium transient experiments in Kir2.1^Δ314-315^ cardiomyocytes suggest that SR Kir2.1 channel function may be an additional important countercurrent during diastole. If oriented as suggested by our voltage-clamp experiments in nuclear vesicles (Fig. 6 and Extended Data Fig. 6), SR Kir2.1 function could explain a fundamental current activity (from the SR lumen to the cytoplasm) giving rise to the countercurrent for SERCA-mediated Ca^2+^ reuptake and, to a lesser extent, RyR-mediated Ca^2+^ release. In Kir2.1^Δ314-315^ cardiomyocytes, Kir2.1 was mis-localized, and the two microdomains showed substantial disorganization. In these conditions, calcium transient dynamics was altered, revealed by both prolonged recovery of the calcium transients and multiple abnormal spontaneous calcium release events. Therefore, data presented here lead us to propose a potential molecular mechanism to explain the spontaneous arrhythmias, skeletal muscle weakness and periodic paralysis reported for patients with ATS1 (refs. ^5,6^). We hypothesize that, in addition to disrupting the Kir2.1–Na_V_1.5 channelosome at the sarcolemma^8,10^, ATS1 also leads to dysfunction of SR Kir2.1 channels, directly altering SR countercurrents, thus disrupting e–c coupling and intracellular Ca^2+^ dynamics. Thus, slower Ca^2+^ reuptake would result in higher cytoplasmic Ca^2+^ lifetime and higher activity of Na^+^/Ca^2+^ exchanger NCX. Consequently, there should be a higher probability of spontaneous Ca^2+^-dependent Ca^2+^ release events and spontaneous APs, similarly to CPVT and heart failure^40,41^. The demonstration of depressed SERCA function, leaky RyRs and increased NCX activity helps us understand the hitherto unexplained phenotypic overlap between ATS1 and CPVT in some patients and the arrhythmias and intermittent paralysis seen in ATS1 and several other skeletal muscle diseases^42–44^.

In accordance with previous reports^1,4,45^, we demonstrate how in vivo trafficking deficiency of one channel component of the Na_V_1.5–Kir2.1 macromolecular complex (in this case, Kir2.1^Δ314–315^) negatively influences the functional localization of the other channel (Na_V_1.5), by disturbing channel trafficking to the plasma membrane. However, our data suggest that the stoichiometry of Kir2.1–Na_V_1.5 interaction at the membrane is unlikely to be 1:1, because voltage-clamp experiments show that more than 85% reduction of I_K1_ density at the membrane caused by cardiac Kir2.1^Δ314–315^ expression is accompanied by only ~40% reduction in *I*_*Na*_ density. Such data highlight the idea that, in mice, like rats and human induced pluripotent stem cell-derived cardiomyocytes^10^, only a fraction of the Na_V_1.5 channels present in a cardiomyocyte share the AP-1-mediated Kir2.1 trafficking pathway as they reach the sarcolemma. Therefore, the data indicate that Na_V_1.5 channels reach the plasma membrane by using more than one alternative pathway^10,15^.

As previous in vitro experiments suggest^16^, expression of Kir2.1 trafficking-deficient mutant channels does not alter the global structure of the mutant monomer, which likely results in the formation of heterotetrameric Kir2.1^WT^–Kir2.1^Δ314–315^ channels translating into a dominant negative effect. As a consequence, decreases in both *I*_*K1*_ and *I*_*Na*_ upon Kir2.1^Δ314–315^ expression are due to alterations in the interaction of Kir2.1 and Na_V_1.5 with common protein partners^8–10,12,46–50^. Here we show that the distribution of one of these partners—that is, the AP1 protein—is compromised by the Kir2.1 ^Δ314–315^ mutation in the ATS1 mouse due to the lack of an AP1 binding site at the C-terminal SY_315_ residues, with consequent accumulation of the protein at the SR. These results lead us to hypothesize that proper Kir2.1–AP1 assembly taking place in the Golgi is necessary for the proper AP1–Na_V_1.5 interaction and trafficking to the sarcolemma. Accordingly, Na_V_1.5 channels may be exported from the Golgi in a signal-dependent manner through an AP1 clathrin adaptor interaction, as suggested previously^10^. As such, trafficking vesicles may carry varying compositions of cargo proteins.

The availability of the ATS1 mouse model with cardiac-specific expression of Kir2.1^Δ314–315^ opens yet unexplored opportunities for the understanding of the molecular mechanisms underlying the disease in patients. However, to date, mutations in *KCNJ2* are the only genetic abnormalities identified in patients with ATS who also present variable cardiac manifestations, despite the fact that approximately 60% of these patients have *KCNJ2* mutations^1,4,5^.

Although structural and functional heart disease has been described in some patients with ATS1 (ref. ^51^), the most common cardiac defects are ECG and rhythm disturbances, including the presence of U waves, mild QT prolongation and conduction abnormalities such as first-degree atrioventricular block and bifascicular block^52^. The arrhythmia burden is usually high, but, surprisingly, patients are mostly asymptomatic^45,51,53^. Nevertheless, cardiac arrest has been documented, and family history of SCD has been identified^51,52^. The AAV-mediated ATS1 mouse model recapitulates many of the above electrical abnormalities, including the high arrhythmia burden and susceptibility to VT/VF.

Conduction and repolarization alterations demonstrated on ECGs of mice expressing Kir2.1^Δ314–315^ are a direct result of reduction of the density of currents generated by both Kir2.1 and Na_V_1.5 channels, as previously suggested^10^. Accordingly, patch-clamp experiments showed reduced in *I*_*K1*_ and *I*_*Na*_ densities with consequent membrane depolarization and APD prolongation. Moreover, the ATS1 mouse phenotype is associated with e–c coupling defects, abnormal spontaneous calcium release, U waves, spontaneous ventricular arrhythmias and increased susceptibility to AF as well as VT/VF induced by intracardiac stimulation.

Notably, we demonstrate that treatment with flecainide, a drug that is used to treat arrhythmias in patients with ATS^54–56^, substantially exacerbates the ATS1 phenotype and leads to both reentrant and multifocal arrhythmia mechanisms in the mouse model. Clearly, the trafficking-deficient Kir2.1 mutation disturbs Na_V_1.5 trafficking, ultimately contributing to further reducing excitability and impulse conduction velocity, establishing the substrate for life-threatening arrhythmias.

**Extended Data Fig. 10:**
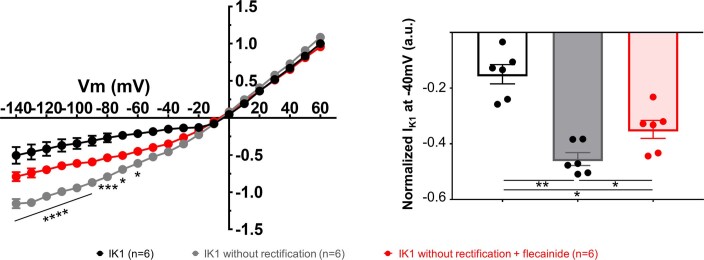
Flecainide induces partial I_K1_ rectification in HEK293 cells. *Left*, current-voltage (I-V) relationships constructed at the end of the test pulses for IK1 with (black, as maximum rectification) and without (grey, as control) polyamine rectification, and after 10 µM flecainide block (red) (*P* < 0.0001 for voltages ≤ −90mV; *P* = 0.0003, for −80mV; *P* = 0.0113, for −70mV; *P* = 0.0342, for −60mV). *Right, g*raphs show the normalized current at −40mV (*P* = 0.0013, IK1 vs IK1 without rectification; *P* = 0.0413, IK1 vs IK1 without rectification + flecainide; and, *P* = 0.0154, IK1 without rectification vs IK1 without rectification + flecainide). Each value is represented as mean ± SEM. Statistical analyses were conducted using Two-way ANOVA and One-way ANOVA test, respectively. *, p < 0.05; **, p < 0.01; ***, p < 0.001; ****, p < 0.0001. Source data

Flecainide has been shown to increase I_K1_ at the sarcolemma by decreasing the Kir2.1 channelʼs affinity for intracellular polyamines^57^. However, because polyamine-induced rectification at the SR vesicles is weaker than sarcolemmal *I*_*K1*_, we surmised that, in the absence of polyamines, flecainide should reduce rather than increase I_K1_ (ref. ^57^). The idea was borne out by inside-out patch experiments in HEK293 cells showing that, in the absence of polyamine-mediated rectification, flecainide blocks I_K1_ (Extended Data Fig. 10). Therefore, in the cardiomyocyte, flecainide should accentuate rectification of the SR Kir2.1 channel, reduce the Ca^2+^ countercurrent and lead to intracellular calcium overload, explaining its pro-arrhythmic effect (Figs. 2 and 3a,b). These results call for caution and revaluation of the widespread use of flecainide in patients with ATS1, particularly those carrying trafficking-deficient Kir2.1 mutations.

As an additional value, this model has permitted us to unravel a potential molecular mechanism for the phenotypic overlap between ATS1 and CPVT in some patients^5,6^ as well as a hitherto unknown actor in such an essential physiological function as e–c coupling in striated muscles. Based on the evidence presented here, we postulate that, in addition to reduced *I*_*K1*_ and *I*_*Na*_^8,10^, some ATS1 mutations also lead to dysfunction of SR Kir2.1 channels, directly altering SR countercurrents and disrupting e–c coupling and intracellular Ca^2+^ dynamics in both intact and permeabilized cells, which results in stress-increased calcium-mediated arrhythmias that mimic CPVT^19,33,35^. To our knowledge, although PIP2 has been extensively proposed as an essential cofactor for plasmalemma Kir2.1 microdomain^58,59^ among others, this phospholipid is only present into the plasmalemma^59^, so Kir2.1 localized in the SR microdomains should not be modulated by this phospholipid. Therefore, we hypothesize that those mutations with alterations in the global structure of the channel or those trafficking-deficient mutations (that is, Kir2.1^∆314-315^) would have alterations in the intracellular calcium dynamics. On the other hand, we cannot exclude the possibility of secondary alterations, such as post-translational modifications in RyRs/SERCA (for example, phosphorylation and oxidation)^60,61^, or even the role of mutant Kir2.1^∆314-315^-preferred interactors^12^ dysregulating the e–c coupling process. However, although further studies will be needed to clarify the precise molecular mechanisms, we have proved the concept that functional alteration of Kir2.1 at the SR modifies the calcium dynamics, resulting in a CPVT-like clinical phenotype. Revealing such mechanisms should lead to more effective targets in the treatment of disorders related to calcium dynamic alterations, including ATS and CPVT.

## Methods

### Study approval

All animal procedures conformed to the guidelines from Directive 2010/63/EU of the European Parliament on the protection of animals used for scientific purposes and to Recommendation 2007/526/EC, enforced in Spanish law under Real Decreto 53/2013. Animal protocols were carried out in accordance with the Centro Nacional de Investigaciones Cardiovasculares (CNIC) Institutional Ethics Committee recommendations and were approved by the Animal Experimentation Committee (Scientific Procedures) of Comunidad de Madrid (PROEX 019/17 and PROEX 111.4/20).

### Mice

WT, 20–25-week-old C57BL/6J male mice were obtained from Charles River Laboratories. Mice were reared and housed in accordance with institutional guidelines and regulations. The mice had free access to food and water. Mouse cardiomyocyte isolation and characterization was done as previously described^8,10,15,62^.

### AAV production and purification

AAV vectors were all produced by the triple transfection method, using HEK293A cells as previously described^1^. AAV plasmids were cloned and propagated in the Stbl3 *Escherichia coli* strain (Life Technologies). Shuttle plasmids *pAAV-empty* vector, *pAAV- KCNJ2c.940-945del6pb (KCNJ2*^*Δ314-315*^*)* and *pAAV-Luc* were derived from *pAcTnT* (a gift from B. A. French) and packaged into AAV-9 capsids with the use of helper plasmids *pAdDF6* (providing the three adenoviral helper genes) and pAAV2/9 (providing rep and cap viral genes), obtained from PennVector. Shuttle vectors were generated by direct cloning (GeneScript) of synthesized fragments from *Nhe*I-*Sal*I into *pAcTnT* cut with the same restriction enzymes.

The AAV shuttle and helper plasmids were transfected into HEK293A cells by calcium-phosphate co-precipitation. A total of 840 µg of plasmid DNA (mixed in an equimolar ratio) was used per HYPERFlask (Corning) seeded with 1.2 × 10^8^ cells the day before. Seventy-two hours after transfection, the cells were collected by centrifugation, and the cell pellet was resuspended in TMS (50 mmol L^−1^ Tris HCl, 150 mmo L^−1^ NaCl and 2 mmol L^−1^ MgCl_2_) on ice before digestion with DNase I and RNaseA (0.1 mg ml^−1^ each; Roche) at 37 °C for 60 minutes. Clarified supernatant containing the viral particles was obtained by iodixanol gradient centrifugation^2^. Gradient fractions containing virus were concentrated using Amicon UltraCel columns (Millipore) and stored at −70 °C.

Determination of AAV vector titer (vg per ml) were carried out by quantitative real-time polymerase chain reaction (PCR) as described^3^. Known copy numbers (10^5^–10^8^) of the respective plasmid (*pAAV-empty* vector, *pAAV-KCNJ2*^*Δ314-315*^ and *pAAV-Luc*) carrying the appropriate complementary DNA were used to construct standard curves.

### AAV injection

Mice were anesthetized with 100 μl of ketamine (60 mg kg^−1^), xylazine (20 mg kg^−1^) and atropine (9 mg kg^−1^) via the i.p. route. Once asleep, animals were located on a heated pad at 37 ± 0.5 °C to prevent hypothermia. A ~4-mm incision was made in the skin to expose the right femoral vein. To increase vessel diameter and facilitate infusion, blood flow was interrupted with a cotton bud for a couple of seconds. Once the vein was dilated, insulin syringe vessel was introduced into the vein, and 3.5 × 10^10^ virus particles were inoculated in a final volume of 50 μl, taking care to prevent introduction of air bubbles. Animals were then sedated with buprenorphine (subcutaneous (s.c.), 0.1 mg kg^−1^) and maintained on the heating pad until recovery. Paracetamol was administered orally for 1 week.

### AAV-mediated gene distribution

Corporal distribution of protein expression was examined as previously described^4^. In brief, 4 weeks after injection, in vivo bioluminescence signal was performed in luciferase control mice, confirming the cardiac expression by similar experiments in ex vivo heart organs from these mice. Infection efficiency was quantified by epifluorescence of whole hearts and microscopic images of cardiac slices in non-infected and AAV-transduced mice.

### ECG recording

#### Surface ECG

Mice were anaesthetized using isoflurane inhalation (0.8–­1.0% volume in oxygen), and efficacy of the anesthesia was monitored by watching breathing speed. Four-lead surface ECGs were recorded, for a period of 5 minutes, from s.c. 23-gauge needle electrodes attached to each limb using the MP36R amplifier unit (BIOPAC Systems).

During offline analysis, lead-II was used for QRS duration using AcqKnowledge 4.1 analysis software. A representative 30-second segment of the recording was averaged to obtain the signal-averaged ECG. QRS duration (before and after flecainide administration, 40 mg kg^−1^, and isoprenaline, 5 mg kg^−1^) was measured as the time interval between the earliest moment of deviation from baseline and the moment when the S wave returned to the isoelectric line. QT duration was measured when the recording returned to the isoelectric line after T wave and was corrected by the Framingham equation^63^.

#### Intracardiac recording

An octopolar catheter (Science) was inserted through the jugular vein and advanced into the right atrium (RA) and ventricle as previously described^7^. Atrial and ventricular arrhythmia inducibility was assessed by application of 12–18 atrial bursts and defined as the occurrence of rapid and fragmented electrograms (lack of regular P waves) with irregular AV-nodal conduction and ventricular rhythm.

### CMR imaging and analysis

During CMR evaluation, animals were anaesthetized with isoflurane and monitored for core body temperature, cardiac rhythm and respiration rate using a CMR compatible monitoring system. In vivo cardiac images were acquired using an Agilent VNMRS DD1 7T MRI system. A k-space segmented ECG-triggered cine gradient-echo sequence was used. After shimming optimization, cardiac four-chamber and left two-chamber views were acquired and used to plan the short-axis sequence. Mice were imaged with the following parameter settings: number of slices, 13; slice thickness, 0.8 mm; gap, 0 mm; matrix size, 256 × 256; field of view, 30 × 30 mm^2^; gating: ECG and respiratory triggered; cardiac phases, 20; averages, 4; effective TE, ~1.8 ms, minimum TR, 7 ms; flip angle, 25°; trigger delay, 2 ms; trigger window, 8 ms; dummy scans, 2.

All CMR images were analyzed using dedicated software (Segment software version 1.9 R3819, http://segment.heiberg.se)^8^. Images were analyzed by two experienced observers blinded to the study allocation. All CMR images were of good quality and could be analyzed. The short-axis dataset was analyzed quantitatively by manual detection of endocardial borders in end-diastole and end-systole, with exclusion of papillary muscles and trabeculae, to obtain both left and right end-diastolic volume, end-systolic volume and ejection fraction.

### Cardiac echocardiography

Transthoracic echocardiography was performed blinded by an expert operator using a high-frequency ultrasound system (Vevo 2100, VisualSonics) with a 40-MHz linear probe. Two-dimensional (2D) and M-mode (MM) echography was performed at a frame rate above 230 frames per second, and pulse wave (PW) Doppler was acquired with a pulse repetition frequency of 40 kHz. Mice were lightly anesthetized with 0.5–2% isoflurane in oxygen, adjusting the isoflurane delivery to try to maintain the heart rate at 450 ± 50 bpm. Mice were placed in the supine position using a heating platform, and warmed ultrasound gel was used to maintain normothermia. A base apex ECG was continuously monitored. Images were transferred to a computer and analyzed off-line using the Vevo 2100 Workstation software (version 5.6.1). For left ventricular (LV) systolic function assessment, parasternal standard 2D and MM, long-axis and short-axis views (LAX and SAX view, respectively) were acquired. LV ejection fraction and chamber dimensions were calculated from these views.

### Optical mapping in isolated hearts

Optical mapping experiments were carried out as previously described^18^. In brief, we used hearts from non-infected, Kir2.1^WT^ and Kir^∆314-315^ mice (20 weeks old). Upon isoflurane anesthesia, the heart was rapidly excised through thoracotomy and subsequently connected to a Langendorff perfusion system to be continuously perfused with warm oxygenated Tyrode’s solution (pH 7.4) with HEPES as buffer, bubbled with 95% O_2_:5% CO_2_. Hearts were placed in a custom-made plastic chamber maintained at 36 ± 1 °C and allowed to equilibrate for 10 minutes. The potentiometric dye Di-4-ANEPPS (Molecular Probes) was added to the perfusated as a bolus to achieve a final concentration of 10 μmol L^−1^. We used an optical mapping system comprised of a custom-made upright 128 × 128-pixel eVolve EMCCD camera (Photometrics) running at 1,000 frames per second. Blebbistatin 10 μmol L^−1^ was used to reduce contraction. Phase, conduction velocity, activation and APD maps were generated in the absence and presence of 10 μmol L^−1^ flecainide using custom-made MATLAB software. Cardiac (right ventricle) stimulation was conducted using a custom-made bipolar electrode (≈700-μm interelectrode distance) connected to a programmed stimulator (Cibertec). Usually, ten pulses (amplitude, 8 V; duration, 2 ms) at 5 Hz followed by ten similar pulses at 25 Hz were applied.

### Cardiac cell isolation

#### Cardiomyocyte isolation

The procedure was adapted from literature^62.64^. In brief, after euthanasia in a CO_2_ chamber, mice were placed in the supine position, and the ventral thoracic region was wiped with 70% alcohol. The heart was quickly removed and incubated at room temperature in Ca^2+^-free Perfusion Buffer (PB; in mmol L^−1^: NaCl, 113; KCl, 4.7; KH_2_PO_4_, 0.6; Na_2_HPO_4_, 0.6; MgSO_4_·7H_2_O, 1.2; NaHCO_3_, 12; KHCO_3_, 10; Phenol Red, 0.032; HEPES, 0.922; taurine, 30; glucose, 5.5; 2,3-butanedione-monoxime, 10; pH 7.4). Fat was cleaned, and the heart was cannulated through the ascending aorta and mounted on a modified Langendorff perfusion apparatus. The heart was then retrogradely perfused (1 ml min^−1^) for 5 minutes at 37 °C with PB. Enzymatic digestion was performed with digestion buffer (DB): PB supplemented with Liberase (0.2 mg ml^−1^), trypsin 2.5% (5.5 mmol L^−1^), DNase (5 × 10^−3^ U ml^−1^) and CaCl_2_ (12.5 µM) for 20 minutes at 37 °C. At the end of enzymatic digestion, both ventricles were isolated and gently disaggregated in 3 ml of DB. The resulting cell suspension was filtered through a 200-µm sterile mesh (SEFAR NITEX) and transferred for enzymatic inactivation to a tube with 12 ml of stopping buffer-1 (SB-1): PB supplemented with FBS (10% v/v) and CaCl_2_ (12.5 µmol L^−1^). After gravity sedimentation for 20 minutes, supernatant was removed, and cardiomyocytes were resuspended in stopping buffer-2 (SB-2) containing lower FBS (5% v/v) for another 20 minutes. Cardiomyocyte Ca^2+^ reintroduction was performed in SB-2 with two progressively increased CaCl_2_ concentrations (0.112 mmol L^−1^ and 1 mmol L^−1^). Cells were resuspended and allowed to decant for 15 minutes in each step, contributing to the purification of the cardiomyocyte suspension.

#### Cardio-fibroblast isolation

After SB-1 addition in the cardiomyocytes isolation protocol, the supernatant was collected and centrifuged at 1,000*g* for 5 minutes, and cells were re-suspended in DMEM/F-12 medium. To perform adherence-mediated fibroblast isolation, cell suspension was seeded in a 24-well culture dish during 30–45 minutes. After that, cells not adhered at the bottom of the dish were removed by gently washing 2–3 times with PBS. For image acquisition, cells were stored in culture for 3 days, changing the medium every day.

### SR vesicles isolation

Nuclei from isolated cardiomyocytes were isolated as previously described^65^. In brief, isolated cardiomyocytes were washed with ice-cold PBS and centrifuged, and the cell pellet was resuspended in an appropriate volume of ice-cold Nuclei Isolation Solution (NIS; in mM: 150 KCl, 250 sucrose, 10 Tris-HCl, 1.4 β-mercaptoethanol, pH 7.3 KOH supplemented with one tablet of complete protease inhibitor cocktail for each 40 ml of NIS; Roche Applied Science, 1697498). Usually, a whole heart was divided in three aliquots, and the cells were resuspended in 250 µl of NIS. Finally, SR vesicles were obtained by resuspending the cell suspension with a 30-gauge syringe 40 times to induce cell lysis and nuclei release.

### Cardiomyocyte detubulation

The detubulation procedure was adapted from Kawai et al.^66^ and Brette et al.^67^. In brief, isolated cardiomyocytes were washed with a bath solution (BS; in mM): 113 NaCl, 5 KCl, 1 MgSO_4_, 1 CaCl_2_, 1 Na_2_HPO_4_, 20 sodium acetate, 10 glucose, 10 HEPES and 5 U L^−1^ insulin, pH to 7.4 NaOH. Cell detubulation was performed after 15 minutes at room temperature in detubulation solution (DS): DS supplemented with formamide 1.5 M and EGTA 5.25 µM. After this, formamide was washed-out with BS.

### Immunofluorescence

#### Isolated cardiomyocytes

Cells were fixed in 4% formaldehyde in PBS at room temperature, shaken gently for 10 minutes, washed with PBS and stored at 4 °C in PBS until use. Cells were then incubated for 10 minutes at room temperature with WGA Alexa Fluor 488 (W11261, Thermo Fisher Scientific, 1/100) under gentle shaking, washed with PBS and re-fixed in 4% formaldehyde in PBS at room temperature with gentle shaking for 15 minutes to avoid dye internalization. Thereafter, cells were prepared for immunofluorescence. Cells were blocked and permeabilized for 90 minutes at room temperature in PBS containing 0.2% Triton X-100 and 10% normal goat serum and incubated overnight at 4 °C with anti-Kir2.1 (1:200, APC-026, Alomone Labs), anti-Nav1.5 (1:50, AGP-008, Alomone Labs), anti-SERCA (1:200, sc-376235, Santa Cruz Biotechnology), anti-RyR2 (1:200, ab2827, Abcam), anti-ankyrin-B (1:200, sc-12718, Santa Cruz Biotechnology) and anti-actinin (1:200, A7732, Sigma-Aldrich). Samples were then incubated for 2 hours at room temperature with secondary antibodies from Thermo Fisher Scientific (Alexa Fluor 488, A-11034; Alexa Fluor 568, A-11075 and A-11031; and Alexa Fluor 680, A-21058; 1/500 in all cases) and mounted in Fluoroshield-DAPI imaging medium (F6057, Merck). In those experiments analyzing the amount of Kir2.1 present in the membrane, non-permeabilized cells were incubated with anti-Kir2.1 (1:100, ab109750, Abcam). Images of individual cardiomyocytes were acquired with a Leica SP8 (LAS-X software version 3.5.7.233225) confocal microscope and HC PL Apo CS2 ×63/1.4 oil objective. Finally, Imaris 7.7.2 and 9.1.2 software (Bitplane) was used for 3D rendering.

#### SR vesicles

The immunofluorescence protocol described above was performed in nuclear samples that were decanted on SuperFrost Plus microscope slides and dried at 37 °C for 1 hour.

### Skeletal myocytes isolation

The flexor digitorum brevis (FDB) was quickly removed and incubated in PB at room temperature. Isolated myocytes from the FDB muscle were obtained by enzymatic digestion with collagenase type I (60 minutes, 1 mg ml^−1^, Sigma-Aldrich) in PB and mechanical dissociation with tip cut pipettes, as described previously^68,69^. Cells were then centrifuged at 40*g* for 3 minutes at room temperature, and medium was changed by fresh PB without enzymes and stored at room temperature until use.

### Calcium dynamics assays in intact cells

Cytosolic Ca^2+^ was monitored as previously described^70,71,72^. In brief, cells were loaded with Fluo-4-AM (Invitrogen) by incubation for 15 minutes in Tyrode’s solution containing 5 μM Fluo-4-AM and 0.02% Pluronic F-127 (Life Technologies) in the dark at room temperature. Cells were allowed to settle to the bottom of the perfusion chamber (RC-26, Warner Instruments) mounted on the stage of an inverted LSM 880 Carl Zeiss confocal microscope (ZEN 2.3 (black) software, version 13.0.0.0) and Plan-Apochromat ×20/0.8 dry before being perfused with the corresponding solution (in mM: 136 NaCl, 5.4 KCl, 1 MgH_2_PO_4_, 10 glucose, 0.9 CaCl_2_ and 5 HEPES, pH 7.4). The same solution was used for the caffeine-induced Ca^2+^ transients. Field stimulation was conducted using a custom-made bipolar electrode (≈700-µm interelectrode distance) connected to a DS2A stimulator (Warner Instruments), and the electrode was attached to a micromanipulator (SensApex). We applied pulses (amplitude, 7 V; duration, 2 ms) at varying frequencies for 10 seconds separated by 15–20-second intervals. All experiments were performed at room temperature. Images were taken using a ×20, NA 0.8 dry objective. Fluo-4-AM fluorescence was detected in line-scan mode (usually 2 ms per scan), with the line drawn approximately through the center of the cell parallel to its long axis. Fluo-4-AM was excited with a blue laser (488 nm), and emission was detected between 505 nm and 605 nm.

### Calcium dynamics assays in permeabilized cells

Spontaneous Ca^2+^ release events in permeabilized cells were monitored similarly to previously described protocols^33^. In brief, once isolated, cardiomyocytes were permeabilized in three steps as follows using different solutions. In between each step, cells were centrifuged at 70*g* for 1 minute at room temperature. The supernatant was discarded, and the pellet was re-suspended into the next solution: (1) washed twice with a Ca^2+^-free Tyrode’s solution to remove Ca^2+^ and avoid Ca^2+^ overload during the permeabilization (in mM: 120 K-Asp, 3 K_2_ATP, 3 MgCl_2_, 0.1 EGTA, 10 Na_2_ phosphocreatine, 10 HEPES and 5 U L^−1^ creatine phosphokinase; pH was adjusted to 7.2 with KOH) for 30 seconds. (2) Myocytes were incubated in a saponin permeabilization solution (in mM: 100 K-Asp, 20 KCl, 3.7 MgCl_2_, 1 EGTA, 10 HEPES and 0.01% saponin; pH was adjusted to 7.2 with KOH) at room temperature during 45–60 seconds. (3) Immediately after permeabilization, the myocytes were centrifuged at 70*g* for 1 minute, and the pellet was re-suspended in internal solution (in mM: 120 K-Asp, 3 K_2_ATP, 3 MgCl_2_, 0.1 EGTA, 10 Na_2_ phosphocreatine, 10 HEPES and 5 U L^−1^ creatine phosphokinase, 8% dextran 40,000 and 0.03 Fluo-4). The [Ca^2+^] free in the internal solution was 40 nM (calculated with MaxChelator, https://somapp.ucdmc.ucdavis.edu/pharmacology/bers/maxchelator/). Then, cells were allowed to settle to the bottom of the perfusion chamber (RC-26, Warner Instruments), and the experiments were carried out within 30 minutes after permeabilization. Ca^2+^ sparks were analyzed by using SparkMaster plugin^73^ previously developed for ImageJ. SR load and Ca^2+^ waves were carried out by using ImageJ version 1.53 and Clampfit software. Total diastolic and systolic calcium were calculated as the total sparks Mass (Amplitude×FullWidth×FullDuration) and the total fluorescence of each line normalized to the mean resting fluorescence (F/F_0_), respectively. Both data were normalized to the area (µm s^−1^) of the line-scan recorded.

### Membrane fractionation and immunoblotting

Ventricles from five mice were extracted and homogenized in ice-cold homogenization medium (HM; 250 mM sucrose, 10 mM HEPES-NaOH pH 7.4, 1 mM EDTA and 1 mM EGTA complemented with a mixture of protease inhibitor (Roche)) using a glass potter homogenizer and then passed through syringe with a 25-gauge needle ten times. The total extract was centrifuged at 1,500*g* for 10 minutes at 4 °C to remove non-disrupted cells and the post-nuclear fraction. Supernatant was supplemented with 3 volumes of HM and centrifugated at 38,400*g* for 2 hours at 4 °C. The crude pellet was processed on an OptiPrep Density Gradient Medium (DGM) ranging from 10% to 30% of iodixanol prepared as described in the datasheet (Alere Technologies). After centrifugation at 130,000*g* overnight (16 hours) at 4 °C on a SW32Ti rotor, four fractions were isolated and further subjected to a 3-hour centrifugation at 170,000*g* at 4 °C on a SW40Ti rotor. Each final pellet was resuspended in RIPA buffer (10 mM PO_4_Na_2_/K buffer pH 7.2, 150 mM NaCl, 1 g/100 ml sodium deoxycholate, 1% Triton X-100 and 1% Nonidet P40) supplemented with a mixture of protease inhibitors (Roche). Identical volumes of each fraction were separated on 8% and 10% SDS-PAGE gels. Primary antibodies were rabbit anti-Kir2.1 (1:200, APC-026, Alomone Labs), mouse anti-calnexin (1:200, MA3-027, Invitrogen), mouse anti-ATPase (1:2,500, ab7671, Abcam) and rabbit anti-Nav1.5 (1:500, AGP008, Alomone Labs). Secondary antibodies were goat anti-mouse/HRP (1:4,000, P044701-2, Agilent Technologies) and goat anti-rabbit/HRP (1:4,000, P044801-2, Agilent Technologies). Immunoblots were carried out using ECL Western Blotting Detector Reagent (RPN2209, Amersham Biosciences) and CP-BU NEW films (EXTVX and AGFA). Kir2.1 protein expression levels were fluorescence quantified by using iBright 1500 (software 1.7.0). In all cases, uncropped images of blots are provided as source data. Protein levels were quantified by densitometry using ImageJ software, version 1.53.

### Patch-clamping in isolated cardiomyocytes

Whole-cell voltage and current-clamp recordings and data analysis procedures were similar to those previously described^9–13^. The external and internal solutions are described in Supplementary Table 1.

Aliquots of cardiomyocytes were placed in a superfusion chamber (RC-26, Warner Instruments) mounted on the stage of an inverted microscope (DMi8, Leica, LAS-X software version 3.0.0.15697). Cells were allowed to settle on the bottom of the perfusion chamber before being perfused with the corresponding solution. After patch rupture, whole-cell voltage or current-clamp recordings were made using an Axopatch 200 B amplifier (Axon Instruments). Pipettes made from borosilicate glass (GD-1, Narishige, OD: 1 mm; ID: 0.6 mm) had resistances of 1–3 MΩ. Series resistance compensation of 80–90% was achieved. All voltage-clamp currents were low-pass filtered at 2 kHz with an analog filter and digitized at 4–10 Hz. For Ca^2+^ current experiments, cells were stabilized for 5 minutes in the whole-cell configuration before starting the voltage-clamp protocols to control for current rundown. Data were recorded using pClamp 10.0 software with Clampex 10.0 program and analyzed with the Clampfit 10.0 program (Axon Instruments). Current amplitudes were normalized to the cell capacitance to account for differences in cell size and expressed as densities (pA/pF).

#### AP recordings

Threshold current was determined using 1-ms pulses at increasing amplitudes (0.2 nA per pulse) and frequency of 1 Hz. Thereafter, APs were evoked by the injection of 1-ms pulses of constant amplitude. APD was measured at 20%, 50%, 70% and 90% of repolarization.

#### Current–voltage relationships—potassium currents

Current–voltage (I–V) relationships for the inwardly rectifying K^+^ current (*I*_*K1*_) were constructed from the current changes produced by a 500-ms voltage-clamp step applied in 10-mV increments from –110 mV to +50 mV from a holding potential of –80 mV at 0.1 Hz. *I*_*K1*_ was calculated by subtracting currents recorded in the absence or presence of 500 µM BaCl_2_

#### Sodium currents

To record the sodium (*I*_*Na*_) I–V relationship, cells were held at –160 mV and stepped for 100 ms from −100 mV up to +5 mV in 5-mV increments at 0.2 Hz. *I*_*Na*_ was measured at the peak. In both cases, leak currents were subtracted using the P/4 protocol.

#### Isolated SR vesicles

After nuclei isolation, the sample was first stained with SYTOX Green 5 µM (S7020, Thermo Fisher Scientific) before experiments for clearer nuclei discrimination. Then, small aliquots were placed in a perfusion chamber (RC-26, Warner Instruments) mounted on the stage of an inverted microscope (DMi8, Leica). Samples were allowed to settle on the bottom of the perfusion chamber before being perfused with the corresponding solution. After patch rupture, whole-vesicle voltage-clamp recordings were made using an Axopatch 200B amplifier (Axon Instruments). Pipettes made from borosilicate glass (GD-1, Narishige, OD: 1 mm; ID: 0.6 mm) had resistances of 6–8 MΩ. Series resistance compensation of 80–90% was achieved. All voltage-clamp currents were low-pass filtered at 2 kHz with an analog filter and digitized at 4–10 Hz. Data were recorded using pClamp 10.0 software with Clampex 10.0 program and analyzed with the Clampfit 10.0 program (Axon Instruments). Whole-vesicle patch-clamping experiments were recorded by using Mg^2+^-free and spermine-free solution on both sides of the patch containing (in mM): 123 KCl, 5 K_2_EDTA, 7.2 K_2_HPO_4_ and 8 KH_2_PO_4_ (pH 7.2 KOH).

#### HEK293 cells

The external and internal solutions are described in Supplementary Table 1. I–V relationships were constructed as indicated from the current changes produced by a 500-ms voltage-clamp step applied in 10-mV increments from –110 mV to +50 mV from a holding potential of –80 mV at 0.1 Hz, as previously reported^10,74^. *I*_*K1*_ was calculated by subtracting currents recorded in the absence or presence of 500 µM BaCl_2_. Inside-out recordings were carried out in HEK293 cells stably transfected with Kir2.1 plated in glass coverslips. Currents were recorded by using those used in SR vesicles: Mg^2+^-free and spermine-free solution on both sides of the patch containing (in mM) 123 KCl, 5 K_2_EDTA, 7.2 K_2_HPO_4_ and 8 KH_2_PO_4_ (pH 7.2 KOH). In those carried out to analyze the flecainide effect of currents without inward rectification, as previously described^75^, pipette solution contained (in mM) 145 KCl, 5 HEPES and 1 CaCl_2_ (pH 7.4 with KOH).

### Statistical analyses

Statistical analyses were performed using GraphPad Prism software versions 7.0 and 8.0. Comparisons were generally made by Student’s *t*-test. Unless otherwise stated, we used one-way or two-way ANOVA for comparisons among more than two groups. For experiments involving individual isolated cardiomyocytes, to account for multiple data points obtained from different cells from one animal, data were analyzed in RStudio software 2022.02.0+443 using a hierarchical three-level and two-level random intercept model^64^, respectively. The model tests for data clustering at each cell isolation and adjusts for any clustering with significance testing. Post hoc pairwise comparisons were carried out for hierarchical testing using Bonferroni’s adjustment. Data are expressed as mean ± s.e.m., and differences are considered significant at *P* < 0.05.

### Reporting summary

Further information on research design is available in the Nature Research Reporting Summary linked to this article.

### Supplementary information

Supplementary Information Supplementary Table 1

Reporting Summary

### Source data

Source Data Fig. 1 Unprocessed western blots

Source Data Fig. 1 Statistical Source Data

Source Data Fig. 2 Statistical Source Data

Source Data Fig. 3 Statistical Source Data

Source Data Fig. 5 Unprocessed western blots

Source Data Fig. 5 Immunofluorescences

Source Data Fig. 5 Statistical Source Data

Source Data Fig. 6 Statistical Source Data

Source Data Fig. 7 Statistical Source Data

Source Data Fig. 8 Statistical Source Data

Source Data Extended Data Fig. 2 Statistical Source Data

Source Data Extended Data Fig. 3 Statistical Source Data

Source Data Extended Data Fig. 5 Statistical Source Data

Source Data Extended Data Fig. 7 Statistical Source Data

Source Data Extended Data Fig. 8 Statistical Source Data

Source Data Extended Data Fig. 9 Statistical Source Data

Source Data Extended Data Fig. 10 Statistical Source Data

## Data Availability

All data supporting the findings in this study are included in the main article and associated files. Source data are provided with this paper.

## Code availability

The custom-made MATLAB software used for the optical mapping experiments analysis will be available upon reasonable request.
