# Supplementary material for: Kir2.1 dysfunction at the sarcolemma and the sarcoplasmic reticulum causes arrhythmias in a mouse model of Andersen–Tawil syndrome type 1
Source: Nat Cardiovasc Res. 2022 Oct 17;1(10):900–17. doi: 10.1038/s44161-022-00145-2 (PMC11358039; doi:10.1038/s44161-022-00145-2)
Supplement: Supplementary file 1 — Supplementary Table 1 [file 44161_2022_145_MOESM1_ESM.pdf]

nature cardiovascular research

Article https://doi.org/10.1038/s44161-022-00145-2

Kir2.1 dysfunction at the sarcolemma and the sarcoplasmic reticulum causes arrhythmias in a mouse model of Andersen–Tawil syndrome type 1

In the format provided by the authors and unedited

SUPPLEMENTARY MATERIAL

Dual Dysfunction of Kir2.1 Underlies Conduction and Excitation-Contraction Coupling Defects Promoting Arrhythmias in a Mouse Model of Andersen-Tawil Syndrome Type 1

Álvaro Macías, PhD,[1] Andrés González-Guerra,[1,#] Ana I. Moreno-Manuel, MSc,[1,#] Francisco M. Cruz, PhD,[1] Lilian K. Gutiérrez, MSc,[1] Nieves García-Quintáns, PhD,[1] Marta Roche-Molina, PhD,[1] Francisco Bermúdez-Jiménez, MD, PhD,[1] Vicente Andrés, PhD,[1,2] María Linarejos Vera-Pedrosa, MSc,[1] Isabel Martínez-Carrascoso, MSc,[1] Juan A. Bernal, PhD,[1,2,*] José Jalife, MD, PhD,[1,2,3,*]

[1]Centro Nacional de Investigaciones Cardiovasculares (CNIC), 28029 Madrid, Spain; [2]CIBER de Enfermedades Cardiovasculares (CIBERCV), Madrid, Spain; [3]Departments of Medicine and Molecular and Integrative Physiology, University of Michigan, Ann Arbor, MI, USA.

Short Title: Dual Function of Kir2.1 and ATS1 arrhythmogenesis

# These authors contributed equally

* These authors jointly supervised this work:

José Jalife, MD, PhD.
Cardiac Arrhythmia Laboratory
Centro Nacional de Investigaciones Cardiovasculares
Melchor Fernández Almagro 3, 28029 Madrid, Spain
Email: jose.jalife@cnic.es
Telephone: +34-91 453 12 00 (Ext. 1512)
FAX: +34-91 453 12 65

Juan A. Bernal, PhD.
Inherited Cardiomyopathies Lab & Head of the Viral Vector Unit (ViVU)
Centro Nacional de Investigaciones Cardiovasculares
Melchor Fernández Almagro 3, 28029 Madrid, Spain
Email: juanantonio.bernal@cnic.es
Telephone: +34-91 453 12 00 (Ext. 3307)
FAX: +34-91 453 12 65

Supplementary Table I: External and internal solutions used in patch-clamp experiments.

	K^+ Currents and Action Potentials		Na^+ Currents	
	Bath solution (mM)	Internal solution (mM)	Bath solution (mM)	Internal solution (mM)
**Product**	pH 7.4 (NaOH)	pH 7.2 (KOH)	pH 7.35 (CsOH)	pH 7.2 (CsOH)
Calcium chloride ($CaCl_2$)	1	1	1	-
Cesium chloride (CsCl)	-	-	132.5	-
Cesium Fluoride (CsF)	-	-	-	135
EGTA	-	10	-	10
Glucose	5.5	-	10	-
HEPES	10	5	20	5
K-Aspartate	-	110	-	-
K_2ATP	-	4	-	-
Potassium Chloride (KCl)	5.4	20	-	-
Magnesium Adenosine-Tri-Phosphate (MgATP)	-	-	-	5
Magnesium Chloride ($MgCl_2$)	1	1	1	-
Niquel (II) Chloride ($NiCl_2$)	-	-	1	-
Sodium Chloride (NaCl)	130	8	5	5
Na_2HPO_4	0.33	-	-	-
