# Supplementary material for: Kir2.1 dysfunction at the sarcolemma and the sarcoplasmic reticulum causes arrhythmias in a mouse model of Andersen–Tawil syndrome type 1
Source: Nat Cardiovasc Res. 2022 Oct 17;1(10):900–17. doi: 10.1038/s44161-022-00145-2 (PMC11358039; doi:10.1038/s44161-022-00145-2)
Supplement: Supplementary file 2 — Reporting Summary [file 44161_2022_145_MOESM2_ESM.pdf]

nature portfolio

Corresponding author(s): Dr. Jose Jalife
Dr. Juan A. Bernal

Last updated by author(s): Aug 29, 2022

Reporting Summary

Nature Portfolio wishes to improve the reproducibility of the work that we publish. This form provides structure for consistency and transparency in reporting. For further information on Nature Portfolio policies, see our Editorial Policies and the Editorial Policy Checklist.

Statistics

For all statistical analyses, confirm that the following items are present in the figure legend, table legend, main text, or Methods section.

n/a	Confirmed	
☐	☒	The exact sample size (n) for each experimental group/condition, given as a discrete number and unit of measurement
☐	☒	A statement on whether measurements were taken from distinct samples or whether the same sample was measured repeatedly
☐	☒	The statistical test(s) used AND whether they are one- or two-sided<br>*Only common tests should be described solely by name; describe more complex techniques in the Methods section.*
☒	☐	A description of all covariates tested
☐	☒	A description of any assumptions or corrections, such as tests of normality and adjustment for multiple comparisons
☐	☒	A full description of the statistical parameters including central tendency (e.g. means) or other basic estimates (e.g. regression coefficient) AND variation (e.g. standard deviation) or associated estimates of uncertainty (e.g. confidence intervals)
☐	☒	For null hypothesis testing, the test statistic (e.g. F, t, r) with confidence intervals, effect sizes, degrees of freedom and P value noted<br>*Give P values as exact values whenever suitable.*
☒	☐	For Bayesian analysis, information on the choice of priors and Markov chain Monte Carlo settings
☐	☒	For hierarchical and complex designs, identification of the appropriate level for tests and full reporting of outcomes
☒	☐	Estimates of effect sizes (e.g. Cohen's d, Pearson's r), indicating how they were calculated

Our web collection on statistics for biologists contains articles on many of the points above.

Software and code

Policy information about availability of computer code

Data collection	Data in this manuscript were collected using ZEN 2.3_Black software from Zeiss (v13.0.0.0) for calcium dynamics experiments, iBright 1500 (software 1.7.0) for protein expression levels, LAS-X software (v3.0.0.15697 and v3.5.7.233225) for patch-clamp and confocal images aquisition, AcqKnowledge 4.1 for ECG signals, Vevo 2100 for echocardiography and Clampex 10.0 from pClamp 10.0 software for electrophysiology.
Data analysis	Data in this manuscript were analyzed using Imaris 7.7.2 and 9.1.2 software (Bitplane) for 3D rendering, NIH ImageJ Fiji v.1.53 for fluorescene images, Segment software v1.9 R3819 for MRI, Vevo 2100 Workstation (v5.6.1) for echocardiography, MATLAB for optical mapping, Clampfit 10.0 from pClamp 10.0 software for electrophysiology and calcium dynamics, and GraphPad Prism software (versions 7.0, 8.0 and 9.0) for data representation analysis. Statistics was performed by using GraphPad Prism software (versions 7.0, 8.0 and 9.0) and RStudio software 2022.02.0+443.

For manuscripts utilizing custom algorithms or software that are central to the research but not yet described in published literature, software must be made available to editors and reviewers. We strongly encourage code deposition in a community repository (e.g. GitHub). See the Nature Portfolio guidelines for submitting code & software for further information.

Data

Policy information about availability of data

All manuscripts must include a data availability statement. This statement should provide the following information, where applicable:

- Accession codes, unique identifiers, or web links for publicly available datasets
- A description of any restrictions on data availability
- For clinical datasets or third party data, please ensure that the statement adheres to our policy

Additional data supporting the findings in this study are included in the main article and associated files. Source data are provided with this paper.

Field-specific reporting

Please select the one below that is the best fit for your research. If you are not sure, read the appropriate sections before making your selection.

☒ Life sciences ☐ Behavioural & social sciences ☐ Ecological, evolutionary & environmental sciences

For a reference copy of the document with all sections, see nature.com/documents/nr-reporting-summary-flat.pdf

Life sciences study design

All studies must disclose on these points even when the disclosure is negative.

Sample size	Sampling size was limited by manual segmentation and tracing. Thus, sample sizes were based similar published studies (Macías et al 2022, PMID: 33624748; Jimenez-Vazquez et al 2022, PMID: 35762211; or, Ponce-Balbuena et al 2018, PMID: 29514831; among many others)<br>All mice hearts, were collected from different litters and on different days to ensure reproducibility.<br>For each experiment and graph the exact experimental number (n) or mice (N) are presented in the figures.
Data exclusions	To exclude outliers from some data sets we performed Grubbs' test, also known as the ESD (extreme studentized deviate) method, to determine whether a given value was a signficant outlier from the rest
Replication	We were able to produce similar results in the independent experiments, as well as in replicates at each condition.
Randomization	We did not compare groups of specimens under different treatments or conditions. When drugs were used, comparisons were made in the same animal.
Blinding	Groups were defined by genotype and data acquisition was not blinded. In some experiments, the same investigator performed all procedures, including animal handling, data analysis and presentation. Nonetheless, for comparisons between groups, data were extracted automatically from the softwares used, so no subjective judgment was involved at any step.

Reporting for specific materials, systems and methods

We require information from authors about some types of materials, experimental systems and methods used in many studies. Here, indicate whether each material, system or method listed is relevant to your study. If you are not sure if a list item applies to your research, read the appropriate section before selecting a response.

Materials & experimental systems

n/a	Involved in the study	
☐	☒	Antibodies
☐	☒	Eukaryotic cell lines
☒	☐	Palaeontology and archaeology
☐	☒	Animals and other organisms
☒	☐	Human research participants
☒	☐	Clinical data
☒	☐	Dual use research of concern

Methods

n/a	Involved in the study	
☒	☐	ChIP-seq
☒	☐	Flow cytometry
☒	☐	MRI-based neuroimaging

Antibodies

Antibodies used	anti-Kir2.1 (1:200, APC-026, Alomone Labs), anti-Nav1.5 (1:50, AGP-008, Alomone Labs), anti-SERCA (1:200, sc-376235, Santa Cruz Biotechnology), anti-RyR2 (1:200, ab2827, Abcam), anti-Ankyrin-B (1:200, sc-12718, Santa Cruz Biotechnology) and anti-Actinin (1:200, A7732, Sigma). Secondary antibodies from Thermofisher (Alexa488, A-11034; Alexa568, A-11075, A-11031; Alexa 680, A-21058; 1/500 in all cases). For plasma fractionation experiments: Primary antibodies were rabbit anti-Kir2.1 (1:200, APC-026, Alomone), mouse anti-calnexin (1:200, MA3-027, Invitrogen), mouse anti-ATPase (1:2500, ab7671, Abcam) and rabbit anti-Nav1.5 (1:500, AGP008, Alomone). Secondary antibodies were goat anti-mouse/HRP (1:4000, P044701-2; Agilent Technologies) and goat

anti-rabbit/HRP (1:4000, P044801-2; Agilent Technologies).
In those experiments analyzing the amount of Kir2.1 present in the membrane non-permeabilized cells were incubated with anti-Kir2.1 (1:100, ab109750, Abcam).

Validation

All antibodies from commercial vendors were validated by the manufacturers on their websites:

-anti-Ankyrin-B (1:200, sc-12718, Santa Cruz Biotechnology) - Anti-Ankyrin B Antibody (2.20) is a mouse monoclonal IgG1 λ raised against the spectrin binding domain of Ankyrin B of human origin It is recommended to detect Ankyrin B of mouse, rat and human origin, by means of WB, IP and IF. Also validated in several publications (PMIDs: 35013173, 33863727, 15258150; among others).

-anti-Actinin (1:200, A7732, Sigma) - α-Actinin is a 100kDa actin-binding protein that is found in muscle as well as non-muscle cells. In smooth muscles, α-actinin is present in dense bodies and plaques whereas in normal skeletal muscles, it is associated with z-discs that define muscle sarcomeres. Monoclonal Anti-α-Actinin (Sarcomeric) shows wide reactivity, as it binds to human, bovine, pig, sheep, rabbit, goat, hamster, cat, rat, mouse, dog, chicken, lizard, snake, frog and fish α-actinin. It has been used in ICC/IF and WB techniques. Also validated in several publications (PMIDs: 29062050, 28870505, 34702928; among others).

-anti-Kir2.1 (1:100, ab109750, Abcam) - Is a rabbit monoclonal [EPR4530] to Kir2.1/KCNJ2. Probably participates in establishing action potential waveform and excitability of neuronal and muscle tissues. Inward rectifier potassium channels are characterized by a greater tendency to allow potassium to flow into the cell rather than out of it. Their voltage dependence is regulated by the concentration of extracellular potassium; as external potassium is raised, the voltage range of the channel opening shifts to more positive voltages. Reacts with Human. Suitable for: ICC/IF, WB, IHC-P. Also validated in several publications (PMID: 30282820, 34702928, 29502106, 30880024; among others).

-Alexa488 (A-11034, Invitrogen) – Is a Goat anti-Rabbit IgG (H+L) Highly Cross-Adsorbed Secondary Antibody, Alexa Fluor™ 488. Invitrogen™ Alexa Fluor 488 dye is a bright, green-fluorescent dye with excitation ideally suited to the 488 nm laser line. Anti-Rabbit secondary antibodies are affinity-purified antibodies with well-characterized specificity for rabbit immunoglobulins and are useful in the detection, sorting or purification of its specified target. Secondary antibodies offer increased versatility enabling users to use many detection systems (e.g. HRP, AP, fluorescence). Species Reactivity against Rabbit. It is has been validated in several publications (PMID: 35981887, 35550346, 35595823, 35920182; among others).

-Alexa568 (A-11031, Invitrogen) - Goat anti-Mouse IgG (H+L) Highly Cross-Adsorbed Secondary Antibody, Alexa Fluor™ 568. Invitrogen™ Alexa Fluor 568 dye is a bright, orange/red-fluorescent dye with excitation ideally suited to the 568 nm laser line. Anti-Mouse secondary antibodies are affinity-purified antibodies with well-characterized specificity for mouse immunoglobulins and are useful in the detection, sorting or purification of its specified target. Secondary antibodies offer increased versatility enabling users to use many detection systems (e.g. HRP, AP, fluorescence). Species Reactivity against Mouse. It is has been validated in several publications (PMID: 35990743, 35949615, 36008558; among others).

-Alexa568 (A-11075, Invitrogen) - Goat anti-Guinea Pig IgG (H+L) Highly Cross-Adsorbed Secondary Antibody, Alexa Fluor™ 568. Fluorescent secondary antibody conjugates are useful in the detection, sorting, or purification of its specified target and ideal for fluorescence microscopy and confocal laser scanning microscopy, flow cytometry, and fluorescent western detection. Species reactivity against Guinea pig. It is has been validated in several publications (PMID: 35760786, 35750685, 35719268; among others).
-anti-calnexin (MA3-027, Invitrogen) - Calnexin, also referred to as IP90, p88 and p90, is an approximately 90 kDa integral membrane protein of the endoplasmic reticulum (ER). Many resident ER proteins act as molecular chaperones and participate in the proper folding of polypeptides and their assembly into multisubunit proteins. MA3-027 detects calnexin from human and mouse tissues. Suitable for: ICC/IF, WB, IHC-P. It is has been validated in several publications (34512542, 32638178, 26107288; among others).
anti-ATPase (ab7671, Abcam) - Is a mouse monoclonal [464.6] to alpha 1 Sodium Potassium ATPase. This is the catalytic component of the active enzyme, which catalyzes the hydrolysis of ATP coupled with the exchange of sodium and potassium ions across the plasma membrane. This action creates the electrochemical gradient of sodium and potassium ions, providing the energy for active transport of various nutrients. Suitable for: IHC-P, WB, ICC. Reacts with: Mouse, Rat, Rabbit, Human, Pig. It is has been validated in several publications (PMID: 33524449, 33740621, 33946369; among others).

-anti-Nav1.5 (ASC-005, Alomone) - Anti-NaV1.5 (SCN5A) (493-511) Antibody (#ASC-005) is a highly specific antibody directed against an epitope of the rat protein. Voltage-gated Na+ channels (NaV) are responsible for myocardial conduction and maintenance of the cardiac rhythm and are essential for the generation of action potentials and cell excitability. Suitable for: ICC/IF, WB, IHC-P. It has been designed to recognize NaV1.5 sodium channel from rat, human, and mouse samples. It is has been validated in several publications (PMID: 35872650, 35465610, 35215332; among others).

-Alexa 680 (A-21058, Invitrogen) - Goat anti-Mouse IgG (H+L) Highly Cross-Adsorbed Secondary Antibody, Alexa Fluor™ 680. Anti-Mouse secondary antibodies are affinity-purified antibodies with well-characterized specificity for mouse immunoglobulins and are useful in the detection, sorting or purification of its specified target. Secondary antibodies offer increased versatility enabling users to use many detection systems (e.g. HRP, AP, fluorescence). This secondary antibody is designed for fluorescent Western blot detection on various near-infrared fluorescence instruments. This antibody can be used for multi-color and multiplexing detection when using other antibodies conjugated to compatible Alexa Fluor™ dyes and wavelengths. Other applications of this antibody include immunofluorescent and fluorescent imaging applications when using instrumentation with appropriate excitation and detection capabilities. Species reactivity against Mouse. It is has been validated in several publications (PMID: 35847859, 35496912, 34973276; among others).

Eukaryotic cell lines

Policy information about cell lines

Cell line source(s)

HEK293 cells. Original commercial source: American Type Culture Collection, USA.

Authentication	None of the cell lines used were authenticated with the exception of their previously described and validated electrophysiological properties .
Mycoplasma contamination	The cell line was tested weekly and was negative for mycoplasma contamination
Commonly misidentified lines (See ICLAC register)	No commonly misidentified cell line was used in the study.

Animals and other organisms

Policy information about studies involving animals; ARRIVE guidelines recommended for reporting animal research

Laboratory animals	Animal experiments were carried out in wild-type 20-25-week-old male C57BL/6 J strain (code: 000664). Animals were reared and housed in accordance with institutional guidelines and regulations. Mice were housed in an air-conditioned room with a 12-h light/12-h dark cycle and free access to water and chow diet.<br>All animal procedures conformed to the guidelines from Directive 2010/63/EU of the European Parliament on the protection of animals used for scientific purposes EU Directive 2010/63EU and Recommendation 2007/526/EC, enforced in Spanish law under Real Decreto 53/2013. Animal protocols were carried out in accordance with the CNIC Institutional Ethics Committee recommendations and were approved by the Animal Experimentation Committee (Scientific Procedures) of Comunidad de Madrid (PROEX 019/17 and PROEX 111.4/20).
Wild animals	The study did not involve wild animals.
Field-collected samples	The study did not involve samples collected from the field.
Ethics oversight	All experimental and other scientific procedures using animals conformed to EU Directive 2010/63EU and Recommendation 2007/526/EC, enforced in Spanish law under Real Decreto 53/2013. Animal protocols were approved by the local ethics committees and the Animal Protection Area of the Comunidad Autónoma de Madrid (PROEX 019/17 and PROEX 111.4/20).

Note that full information on the approval of the study protocol must also be provided in the manuscript.
