# Supplementary figures and images for: Kir2.1 dysfunction at the sarcolemma and the sarcoplasmic reticulum causes arrhythmias in a mouse model of Andersen–Tawil syndrome type 1
Source: Nat Cardiovasc Res. 2022 Oct 17;1(10):900–17. doi: 10.1038/s44161-022-00145-2 (PMC11358039; doi:10.1038/s44161-022-00145-2)

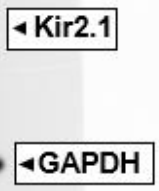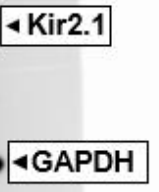

Supplement: Supplementary file 3 — Unprocessed western blots [file 44161_2022_145_MOESM3_ESM.pdf]
