# Supplementary material for: Kir2.1 dysfunction at the sarcolemma and the sarcoplasmic reticulum causes arrhythmias in a mouse model of Andersen–Tawil syndrome type 1
Source: Nat Cardiovasc Res. 2022 Oct 17;1(10):900–17. doi: 10.1038/s44161-022-00145-2 (PMC11358039; doi:10.1038/s44161-022-00145-2)
Supplement: Supplementary file 7 — Unprocessed western blots [file 44161_2022_145_MOESM7_ESM.pdf]

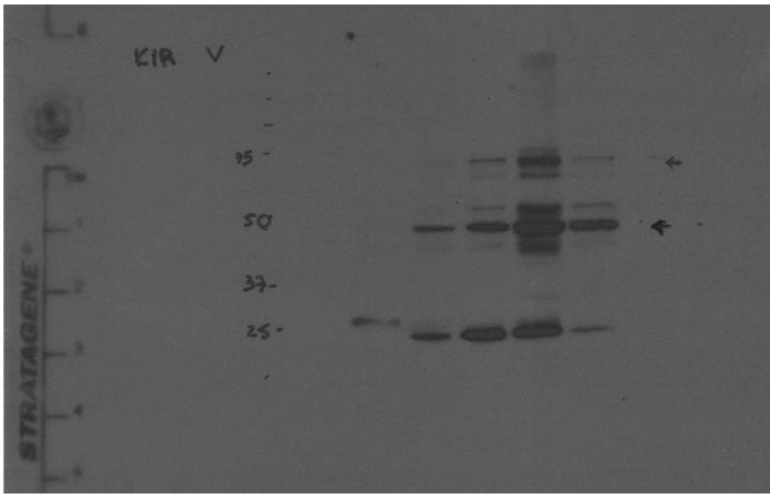

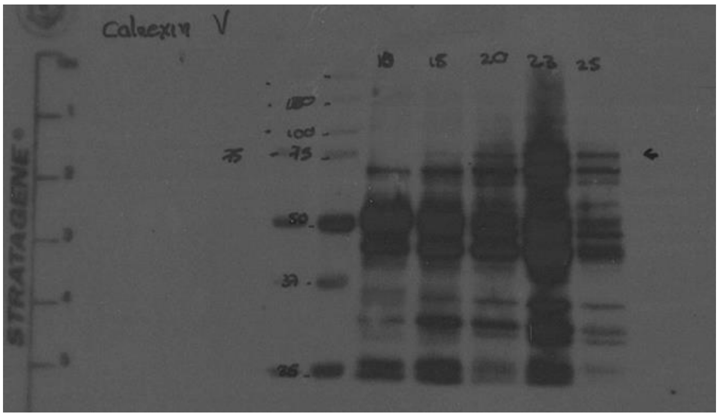

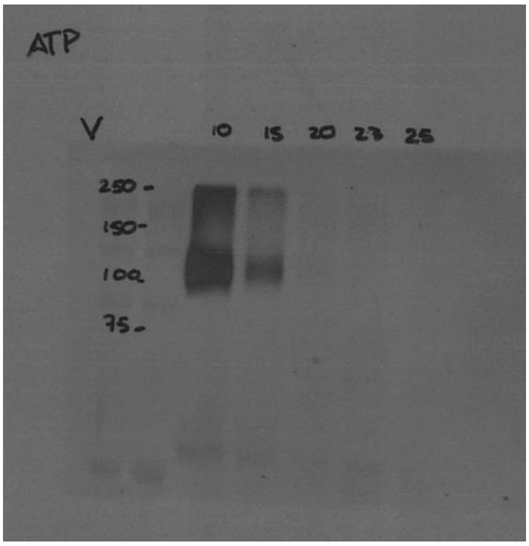

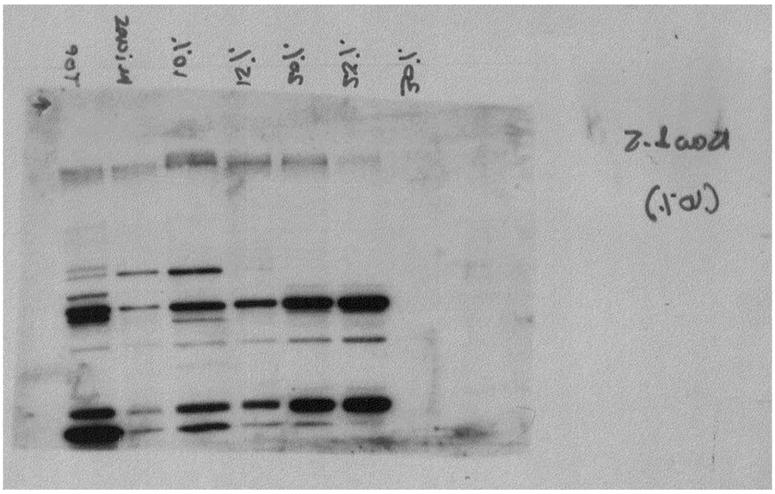

Note that labels appear in mirror format since the gels were loaded in the reverse order to those finally used in the paper
